# Factors Influencing Adherence to Digital Lifestyle Interventions for Adolescents: Systematic Review and Meta-Analysis of Attrition

**DOI:** 10.2196/84822

**Published:** 2026-09-30

**Authors:** Nikolaos Boumparis, Olivia Studhalter, Philippe de Riedmatten, Inci Derya Yücel, Kleio Koutra, Katrina Champion, Ana Molina-Barceló, Teresa de Pablo-Pardo, Haridimos Kondylakis, Michael Patrick Schaub, Andreas Triantafyllidis, Severin Haug

**Affiliations:** 1 Swiss Research Institute for Public Health and Addiction, University of Zurich, Konradstrasse 32, Zürich, 8005, Switzerland, 41 44 4481173; 2 Institute on Drug Abuse, Toxicology and Pharmaceutical Science, Ege University, Bornova/İzmir, Turkey; 3 Hellenic Mediterranean University Social Applied Research and Social Work Lab, Heraklion, Greece; 4 The Matilda Centre for Research in Mental Health and Substance Use & School of Public Health, University of Sydney, Sydney, Australia; 5 Cancer and Public Health Research Unit, Foundation for the Promotion of Health and Biomedical Research, Valencia, Spain; 6 Computer Science Department, University of Crete & Institute of Computer Science, Heraklion, Greece; 7 Centre for Research and Technology Hellas, Thessaloniki, Greece

**Keywords:** adherence, digital health interventions, adolescents, health promotion, systematic review

## Abstract

**Background:**

Intervention studies frequently report that fewer than half of adolescent participants complete digital health interventions as intended, yet a systematic synthesis of the factors driving this low adherence across lifestyle domains is lacking. Understanding these factors is essential for informing the design of interventions that can sustain adherence and achieve meaningful behavioral outcomes.

**Objective:**

This systematic review identified and analyzed factors influencing adherence to digital health interventions targeting key adolescent lifestyle domains: physical activity, dietary habits, obesity management, alcohol consumption, and tobacco use.

**Methods:**

We systematically searched PubMed, PsycINFO, Embase, and CINAHL in March 2024, with the search updated in June 2026, for studies of digital interventions targeting adolescents (10‐19 years). Studies were included if they reported adherence outcomes. Data on intervention characteristics, user-related factors, and adherence outcomes were extracted and narratively synthesized using a framework distinguishing intervention-related and user-related factors across 3 evidence types (statistical, descriptive, and qualitative). Study-level attrition was additionally pooled in a random-effects meta-analysis.

**Results:**

A total of 116 studies, comprising 46,029 participants, were included. The average completion rate (binary measure) was 66.9% (SD 26%), while average adherence to individual intervention components was 55.2% (SD 25.5%). A meta-analysis of attrition across 108 comparisons yielded a pooled dropout of 16.9% (95% CI 13.6‐20.9), with very high between-study heterogeneity and no significant moderators. Consistently reported facilitators included personalization, user-friendly design, reminders, goal setting, gamification, and human support (particularly health coaching and parental involvement). The most common barriers were technical difficulties and poor system usability. Evidence regarding the effects of intervention duration and social components on adherence was inconsistent.

**Conclusions:**

Given that adolescents frequently underuse digital health interventions, completing only about half of intervention components on average, the effectiveness of these interventions is fundamentally constrained by adherence challenges. Adherence is primarily driven by user-centered design features and robust technical performance, while the impact of social and user-related factors varies by health domain and study context. Future research should adopt standardized adherence frameworks and reporting standards to enable meaningful cross-study comparisons and the development of evidence-based engagement strategies.

## Introduction

Digital health interventions, including web-based platforms, mobile apps, wearable device interfaces, and technology-integrated school programs, are increasingly used to promote behavioral change among adolescents. The proliferation of these interventions is driven by several factors: adolescents’ high affinity for digital technologies, the scalability and cost-effectiveness of digital delivery models, and the growing recognition that health-promoting behaviors established during adolescence serve as foundational determinants for preventing noncommunicable diseases across the lifespan [1,2]. Recent meta-analytic evidence, however, suggests that the effects of digital health interventions on adolescent lifestyle behaviors are small and often short-lived, with limited sustained impact on outcomes such as physical activity, dietary behavior, and substance use [3]. Despite these modest effect sizes, the development and deployment of digital health interventions for adolescents continues to accelerate, propelled by commercial interest, low-cost dissemination through social media channels, and the accessibility of app-based delivery for a population that increasingly turns to digital solutions before seeking in-person support [4]. This trajectory makes it important to understand why these interventions often fail to achieve their intended outcomes.

A key factor underlying the limited effectiveness of digital health interventions is suboptimal adherence. Research consistently shows that many users discontinue digital health apps prematurely, directly compromising intervention efficacy and therapeutic outcomes [5,6]. For the purposes of this review, we adopt the conceptual framework proposed by Perski et al [7], which distinguishes between behavioral engagement (the extent of usage, ie, the amount of the intervention that is used) and experiential engagement (the subjective experience of using the intervention, eg, interest, attention, and affect). We focus specifically on the behavioral dimension of engagement, hereafter referred to as adherence, defined as the extent to which adolescents engage with digital interventions as intended by the developers, including frequency of use, completion of prescribed activities, and sustained participation over the intended duration. We do not systematically assess subjective engagement experiences (eg, satisfaction and perceived helpfulness), though these are noted where reported in included studies. This focus aligns with established frameworks for measuring engagement in digital behavior change interventions [8,9]. The challenge of sustaining adherence is particularly pronounced among adolescents, who may require engagement strategies tailored to their developmental stage to maintain consistent participation [4].

Adherence to digital health interventions has been extensively studied in adult populations, particularly in the context of mental health and chronic disease management, where systematic investigations have identified key predictors including user motivation, intervention design characteristics, and technology usability factors [5,10]. However, adolescents differ from adults in developmentally significant ways that may affect their engagement with digital technologies. Adolescents are navigating a critical period of identity formation and social development, during which cognitive functions including sustained attention, impulse control, and decision-making capacity are still maturing [11,12]. These developmental characteristics suggest that determinants of adherence identified in adult populations may not directly apply to adolescent users, necessitating a population-specific evidence synthesis.

Several categories of factors have been proposed as influencing adherence to digital health interventions among adolescents. At the individual level, intrinsic motivation, self-efficacy beliefs, and developmental readiness for behavioral change have been identified as potential determinants, while household-level factors such as parenting practices, family support structures, and socioeconomic considerations may also play a role [13,14]. At the intervention level, technical design elements including personalization algorithms, automated reminder systems, intuitive user interfaces, goal-setting mechanisms, and self-monitoring capabilities have been proposed as critical determinants of sustained engagement [15,16]. Various strategies to enhance engagement have also been suggested, including participatory co-design approaches, adaptive personalization protocols, and just-in-time intervention delivery [4]. However, the empirical evidence for these factors has not been comprehensively synthesized for adolescent populations specifically.

While previous systematic reviews and meta-analyses have examined the overall effectiveness of digital health interventions for adolescents [2,17] and investigated determinants of adherence in mobile health apps among general adult populations [5,18], no comprehensive synthesis exists that specifically focuses on factors influencing adherence within digital health interventions targeting lifestyle behaviors among adolescents. This represents a significant knowledge gap. Without such a synthesis, researchers and developers lack an evidence base to inform the design of interventions that can sustain adolescent adherence, which is a prerequisite for achieving meaningful behavioral outcomes. Given that average adherence rates in digital health interventions are around 50%, even modest improvements in sustained adherence could substantially enhance intervention effectiveness at the population level.

This systematic review therefore aims to identify, categorize, and synthesize the empirical evidence on factors that influence adherence to digital lifestyle interventions among adolescents aged 10‐19 years. By examining evidence across multiple health domains, namely physical activity, dietary habits, obesity management, alcohol consumption, and tobacco use, we provide a cross-domain synthesis that reveals both consistent determinants and domain-specific patterns. The findings are intended to generate actionable insights for researchers, intervention developers, and policymakers working to design digital health interventions that achieve sustained adherence among adolescent populations.

## Methods

### Identification of Studies

The review was guided by the following Population-Exposure-Outcome (PEO) framework: Population: adolescents aged 10‐19 years; Exposure: digital health interventions (web-based, app-based, or text message–based) targeting one or more of 5 lifestyle health domains (physical activity, dietary habits, obesity management, alcohol consumption, and tobacco use); Outcome: adherence to the digital intervention, operationalized as the extent of use relative to intended use parameters specified by intervention developers, including completion rates and component-level adherence metrics.

A comprehensive literature search was conducted across 4 electronic databases: PubMed, PsycINFO, CINAHL, and Embase in March 2024. No restrictions were applied regarding publication date, language, or geographic region. The search strategy was developed iteratively by one researcher (NB) and used combinations of controlled vocabulary terms (MeSH, Emtree, CINAHL Subject Headings, and PsycINFO Thesaurus terms) and free-text keywords across 3 key concepts: adherence or engagement, digital health interventions, and specific health domains (physical activity, diet, obesity, alcohol, and tobacco). While our inclusion criteria encompassed internet-, computer-, and smartphone-based interventions, the term “digital interventions” is used throughout this review for the sake of brevity. The initial screening process involved reviewing titles and abstracts, followed by a detailed assessment of full-text articles for studies that potentially met our inclusion criteria. Two researchers from our team independently performed the search and screening processes, resolving any discrepancies through discussion until consensus was reached (OS and PdR). Discrepancies at each stage were resolved through discussion between the two reviewers. In cases where consensus could not be reached, the senior members of our team (NB and SH) were consulted. The complete search strategy, including detailed search strings, can be found in Multimedia Appendix 1. The search was updated on June 22, 2026, to capture studies published since the original search. Using the identical search strings across the same 4 databases (PubMed, PsycINFO, CINAHL, and Embase), we retrieved records covering the period from March 2024 to June 2026. The deduplicated updated records were screened against the original eligibility criteria; title and abstract screening, full-text screening, and data extraction of the newly included studies were performed by 2 researchers (IDY and NB), with discrepancies resolved through discussion. This systematic review was conducted and reported in accordance with the PRISMA (Preferred Reporting Items for Systematic Reviews and Meta-Analyses) 2020 guidelines [19].

### Eligibility Criteria

Studies examining digital interventions targeting adolescents aged 10‐19 years (as defined by the World Health Organization [WHO]) were required to focus on at least one of the following 5 specified health domains: physical activity, diet, obesity management, alcohol use, and tobacco use. Studies including some participants older than 19 years were retained when the intervention primarily targeted adolescents or youth and the sample mean age was within or very close to the 10‐ to 19-year adolescent age range. These 5 health domains were selected based on their established role as modifiable lifestyle factors associated with the prevention of cancer. The selection was informed by the SUNRISE project (Sustainable Interventions and Healthy Behaviours for Adolescent Primary Prevention of Cancer with Digital Tools; Horizon Europe, Grant Agreement Number 101136829), within which this review was conducted.

These domains were selected based on their established links with chronic disease (eg, cancer, cardiovascular disease, diabetes, and so on) prevention and represent modifiable lifestyle factors that have been shown to reduce cancer risk throughout life. Eligible studies were required to provide qualitative or quantitative data on the intended use parameters or actual adherence patterns with digital intervention components as defined by the developers.

No restrictions were applied regarding publication date or language. Interventions had to implement digital health promotion strategies as the primary therapeutic component; studies in which digital strategies merely supplemented in-person interventions were deemed ineligible. Intended use was operationally defined as the minimum level of digital platform interaction specified by the intervention developers as necessary for achieving therapeutic adherence. Actual use, on the other hand, encompassed the adherence patterns that were empirically measured within the study population. Studies combining multiple intervention modalities, such as automated messaging platforms integrated with counseling calls, were included, provided adherence measures could be isolated and attributed specifically to digital components. Supplementary elements were categorized as “personal support.”

To focus on primary empirical evidence, we excluded systematic reviews and meta-analyses. Single-session interventions were excluded as they preclude meaningful longitudinal adherence assessments, resulting in universal participation rates that provide limited insight into sustained adherence patterns. Studies that used digital platforms solely for communication (eg, email-based coaching or video consultation sessions) were excluded as they represent interpersonal rather than digital behavioral intervention modalities. Additionally, studies that primarily targeted families or other populations rather than adolescents directly were deemed to be outside the scope of this review.

### Data Extraction

Data from studies included in the initial systematic literature search were extracted independently by 2 researchers (OS and PdR), while data for studies identified through the updated systematic search were extracted independently by 2 researchers (IDY and NB). Any discrepancies were resolved through discussion. The following information was extracted for each included study: general study characteristics, study population characteristics, intervention characteristics, and information on intervention use.

General study characteristics included the country, study design, and health domain. Study population characteristics comprised mean age, gender distribution (in %), target group (high school students, university students, and others such as adolescents not enrolled in formal education, those in vocational training programs, community-based youth groups, or clinical populations), intervention setting (online, school-based, home-based, community-based, or clinic-based), presence of clinical diagnosis, number of participants, and inclusion and exclusion criteria. Intervention characteristics included the intervention name, delivery type (web-based, through an app, and through text messages), presence and type of personal support, and provision of participant incentives. Information on intervention use encompassed study duration, number of modules, intended use, actual participant adherence, and attrition rate. Additionally, we extracted data on intervention effectiveness and all reported factors influencing adherence. These factors were classified as either intervention-related, that is, modifiable elements of the intervention design, or user-related, that is, inherent characteristics of the participants. The synthesis of studies identified through the initial systematic search was conducted by OS and reviewed by PdR, whereas the synthesis of studies identified through the updated systematic search was conducted by IDY and reviewed by NB. Factor categorizations and evidence classifications were cross-checked against the original study manuscripts. Any disagreements were resolved through discussion with the senior team members (NB and SH).

### Data Synthesis

To guide our synthesis, we adopted a framework informed by the conceptualization of engagement with digital behavior change interventions proposed by Perski et al [7], which distinguishes between behavioral engagement (extent of usage) and experiential engagement (subjective experience). This review focuses on behavioral engagement, operationalized as adherence: the degree to which participants used the intervention as intended by developers. Adherence was captured through two primary metrics where reported: (1) completion rate, defined as the proportion of participants who completed the entire intervention program (binary measure), and (2) component adherence, defined as the mean proportion of individual intervention components completed by participants (continuous measure). The factors influencing adherence were categorized as intervention-related (modifiable elements of intervention design and delivery) or user-related (participant characteristics), and further classified by evidence type: statistical (inferential testing, *P*<.05), descriptive (quantitative patterns without significance testing), or qualitative (interviews, focus groups, and open-ended responses).

As a first step, the identified studies were categorized according to their respective health domains. In the second step, factors influencing adherence reported in the reviewed articles were qualitatively evaluated and summarized in individual tables for each health domain (Tables S1-S6 in Multimedia Appendix 1 [20-110]). These factors were then categorized into intervention-related factors that acted as either facilitators or barriers to adherence, as well as user-related factors that either facilitated or hindered adolescents’ adherence to digital interventions. In the third step, the factors were analyzed based on 3 types of evidence: statistical significance (findings derived from inferential statistical analyses with *P*<.05), descriptive evidence (quantitative patterns identified through descriptive statistics without formal significance testing), and qualitative evidence (findings from interviews, focus groups, and open-ended survey responses). The compiled tables present an integrated view of these 3 evidence types to provide a comprehensive understanding of factors influencing adherence.

To complement the narrative synthesis, we conducted a quantitative synthesis of attrition. For each intervention arm reporting sufficient data, we calculated the proportion of enrolled participants lost during the intervention and meta-analyzed the logit-transformed proportions using a random-effects model, with the between-study variance estimated using restricted maximum likelihood and CIs calculated using the Knapp-Hartung adjustment [111]. Summary estimates and CIs were backtransformed to the proportion scale. Heterogeneity was quantified using τ², *I*², and a 95% prediction interval. Robustness was examined in sensitivity analyses using a binomial-normal generalized linear mixed model with a logit link [112,113], and a 3-level random-effects model in which estimates from multiple intervention arms were nested within studies [114]. Subgroup analyses and random-effects meta-regression examined health domain, delivery modality, level of personal support, provision of incentives, intervention duration, and mean participant age as potential moderators. Potential small-study effects were explored using funnel plots and an Egger-type regression test [115] because conventional funnel-plot methods may be misleading in meta-analyses of proportions; these analyses were considered exploratory. Reported adherence determinants were not quantitatively pooled because of substantial heterogeneity in their measurement. Instead, we summarized the direction and consistency of each determinant across health domains and evidence types and reported this synthesis in accordance with the Synthesis Without Meta-analysis (SWiM) reporting guideline [116]. Analyses were conducted in R (version 4.5.3; R Core Team) [117] using the *metafor* package [118].

## Results

### Selection and Inclusion of Studies

The original search of the electronic databases was performed on March 12, 2024, and yielded 6831 records. After removal of duplicates, 5385 records remained for title and abstract screening, and the full texts of 408 articles were subsequently examined, of which 85 records (82 studies) [20-76,119-146] were included. The search was updated on June 22, 2026, across the same 4 databases and identified 2298 records; after removal of 413 duplicates, 1885 records were screened by title and abstract, of which 101 were sought for retrieval and assessed as full texts. Of these, 63 were excluded (10 conference posters or abstracts, 21 for an ineligible population, 19 because the digital component was not the primary intervention, 6 for a health domain outside the review scope, 3 for reporting no adherence or engagement data, and 4 for an ineligible study design), leaving 38 newly included records. These corresponded to 34 additional study entries after publications reporting on the same participant cohort were combined, yielding a total of 116 included studies [20-69,71-109,119-144,147]. Figure 1 visualizes the selection process and reasons for exclusion for both the original and updated searches.

**Figure 1.**
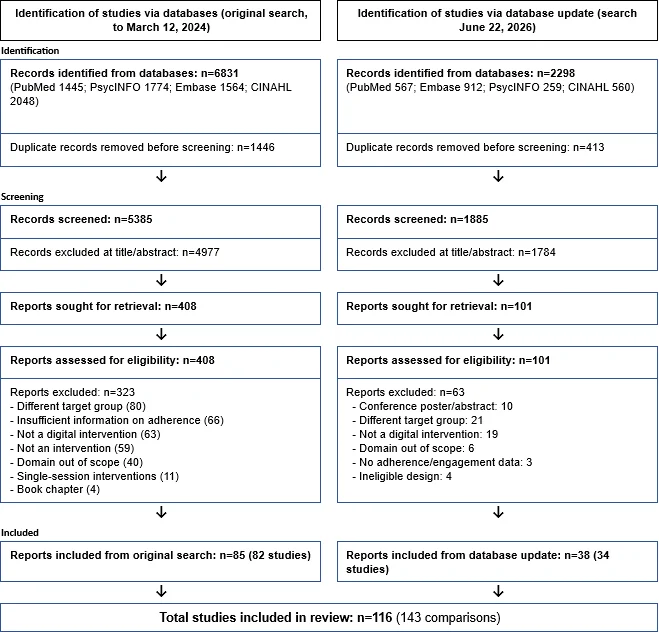
Flowchart for inclusion of studies.

### Characteristics of Included Studies

The 116 included studies comprised several designs: 77 (66.4%) were randomized controlled trials (RCTs) [24,26,28,31,34,37-41,43,46,48,49,51,53-55,57-60,63,66,68,69,71-77,79,81-83,85,87,92-94,96-98,100-107,109,119-121,123-129,131-140,143,144,147], 36 (31%) were quasi-experimental studies [20-23,25,27,29,30,32-34,36,42,44,45,47,50,52,61,62,64,65,67,78,80,84,88-91,99,108,122,130,141,142], and 3 (2.6%) were observational studies [56,86,95].

Geographically, the included studies spanned 26 countries across 5 continents, with persistent regional disparities. The United States remained the single largest contributor, accounting for 43.1% (50/116) studies, followed by Canada, Spain, and Australia. European studies again formed a substantial group, and the updated search broadened the geographic spread by adding studies from countries not previously represented, including Brazil, Uzbekistan, Vietnam, Taiwan, India, Turkey, and Portugal.

The analysis included 143 comparisons from the 116 included studies, as 23 studies [22,26,35,39,40,52,57,67,81,82,85,87,89,91,97,104,107,108,126,129,134,136,147] contained more than one intervention group relevant to our systematic review (21 contributed 2 intervention arms and 2 [85,108] contributed 4 intervention arms). Studies published on the same participant cohort were combined into single entries in the tables and handled as one study. The median intervention duration was 84 (IQR 42-135; range 2-730) days, with 27/143 (18.9%) comparisons lasting 1‐30 days, 54/143 (37.8%) lasting 1 to 3 months, 44/143 (30.8%) lasting 3 to 12 months, and 4/143 (2.8%) lasting 1 year or longer. Fourteen comparisons out of 143 comparisons (9.8%) did not report intervention duration. Of the 143 comparisons, 79 (55.2%) provided no incentives to participants, 44 (30.8%) offered monetary or material rewards such as cash, gift cards, vouchers, prize draws, or a retained device, and 20 (14%) offered nonmonetary incentives such as academic credit or gamified points. A detailed description of the study characteristics can be seen in Tables S7-S18 in Multimedia Appendix 1 [20-110,119-144,147].

### Characteristics of Study Populations

The included studies comprised a total of 46,029 participants summed across intervention and control arms, with a median of 52.5 (IQR 28-232; range 10-5260) participants per study. Of the 142 comparisons that reported exact participant numbers, 91 (64.1%) included fewer than 100 participants, 33 (23.2%) had between 100 and 500 participants, 9 (6.3%) included 500 to 1000 participants, and 9 (6.3%) had more than 1000 participants. The overall mean age was 14.48 (SD 2.07) years. Across the studies reporting participant sex, female participants comprised 59.1% of the combined sample (115 [20-69,71-87,90-109,119-144,147] of 116 studies reported sex), essentially unchanged from the original cohort’s 59.2.

The great majority of studies enrolled adolescents with no prior clinical diagnosis. Among studies recruiting clinically defined populations, obesity or overweight remained the most common, followed by childhood cancer, congenital or coronary heart disease, type 1 diabetes mellitus, and juvenile idiopathic arthritis. The update introduced clinical populations absent from the original synthesis, including single studies of chronic kidney disease, cystic fibrosis, elevated blood pressure or cardiovascular risk, and adolescents recruited through mental health services, together with a larger group of studies enrolling adolescents who used nicotine or electronic cigarettes.

### Characteristics of Digital Interventions

Across the 143 reviewed comparisons, 71 (49.7%) were delivered through location-independent digital platforms accessible via smartphones or personal devices, 33 (23.1%) were school-based, 19 (13.3%) were home-based, 11 (7.7%) were clinic-based, 5 (3.5%) were community-based, and 4 (2.8%) combined settings such as summer camp plus online or school plus mobile delivery.

Regarding delivery mode, 51 (35.7%) comparisons were app-based and 50 (35%) were internet- or web-based, together accounting for roughly 7 in 10 interventions; 13 (9.1%) paired a wearable activity tracker or accelerometer with a smartphone or web platform, 11 (7.7%) relied exclusively on text messaging, 9 (6.3%) were computer- or software-based, 8 (5.6%) used a mixed multimodal approach, and 1 (0.7%) delivered exercise through immersive virtual reality.

Personal support beyond initial recruitment was provided in 72 comparisons (50.3%), most commonly through research-team contact, health-professional input, or coaching, while the remaining 71 (49.7%) offered no personal support or relied solely on automated prompts and reminders.

Effectiveness was determined based on whether studies demonstrated statistically significant improvements in their prespecified primary outcomes, distinct from adherence metrics; 52.8% (47/89) of the interventions evaluated for effectiveness were reported as effective. The mean intervention completion rate across the 68 comparisons reporting it was 66.9% (SD 26%), and component adherence averaged 55.2% (SD 25.5%) across the 66 comparisons that reported it, with the remaining comparisons not reporting full completion or quantitative adherence data.

### Characteristics of Health Domains

As displayed in Tables S7-S18 in Multimedia Appendix 1 [20-110,119-144,147], the included studies were categorized into 5 distinct health domains based on their intervention focus (physical activity, diet, obesity, alcohol use, and tobacco use), with an additional category for multicomponent lifestyle interventions that targeted multiple health domains. Of the 116 included studies [20-69,71-109,119-144,147], 31 (26.7%) [61-69,71-76,98-109,141-143,147] were multicomponent lifestyle interventions, 32 (27.6%) [20-33,77-86,119-125,144] focused on physical activity, 19 (16.4%) [34-48,127-130] targeted obesity, 14 (12.1%) [55-57,90-94,131-136] targeted tobacco use, 10 (8.6%) [49-54,87-89,126] focused on diet, and 10 (8.6%) [58-60,95-97,137-140] addressed alcohol use.

Within the 31 multicomponent lifestyle interventions, the most common combination was diet and physical activity (12 studies, 38.7% [61,62,64-66,68,74,76,102,104,107,142]), followed by alcohol and tobacco (5, 16.1% [63,69,71,72,143]), diet and obesity (4, 12.9% [100,101,105,147]), all 4 of alcohol, diet, physical activity, and tobacco simultaneously (4, 12.9% [73,75,103,109]), diet, physical activity and obesity (3, 9.7% [67,98,99]), physical activity and obesity (2, 6.5% [106,108]), and diet, physical activity, and tobacco (1, 3.2% [141]).

Across the health domains, dietary interventions showed the highest mean intervention-completion rate (83.9%, SD 21%) and alcohol-focused interventions the lowest (48.4%, SD 35.4%); for component adherence, dietary interventions again showed the highest mean rate (63.7%, SD 23%, essentially level with physical activity interventions at 62.8%), while obesity interventions showed the lowest (45.9%, SD 22.5%). These domain-level figures rest on small and unevenly distributed numbers of reporting comparisons (n=4‐24 per domain) and on widely overlapping ranges, and are therefore indicative rather than definitive. In the meta-analysis of attrition, pooled dropout was lowest for physical activity and highest for alcohol interventions, as detailed in the “Quantitative Synthesis of Attrition” section.

The findings presented below are organized according to 3 distinct levels of evidence synthesis: statistical evidence, descriptive evidence, and qualitative evidence. Where individual studies did not generate all 3 types of evidence, only the categories of evidence that were empirically identified are reported and analyzed in the subsequent sections. For a detailed description of all the reported quantitative adherence values, please refer to Tables S8, S10, S12, S14, S16, and S18 in Multimedia Appendix 1 [20-110,119-144,147].

### Quantitative Synthesis of Attrition

Across the included studies, 108 comparisons (88 studies [20,23-30,32-34,37-42,46,49-51,53-60,62,64,65,67,69,72-83,85,87-90,92-95,97-102,104,106-110,119-125,128-130,132-134,136-138,140,142,143,147]; 30,956 participants) reported extractable attrition data. The pooled attrition proportion was 16.9% (95% CI 13.6-20.9). Heterogeneity was very high (*I*^2^=98.3%; τ^2^=1.51), and the 95% prediction interval was correspondingly wide (1.7-70.3), indicating that attrition varied substantially across study contexts. Estimates were robust to model choice (exact binomial-normal model: 14.3%, 95% CI 11.1-18.2; 3-level model accounting for the nesting of multiple intervention arms within studies: 17.3%, 95% CI 13.6-21.7).

Pooled attrition was lowest for physical activity (14.1%, 95% CI 9.9-19.7; k=32), diet (14.9%, 95% CI 6.2- 31.5; k=9), and obesity (15.1%, 95% CI 7.3-28.4; k=14) interventions, intermediate for multicomponent interventions (17.9%, 95% CI 11.1-27.8; k=31), and highest for alcohol (25.3%, 95% CI 7.0-60.5; k=8) and tobacco (21.4%, 95% CI 11.9-35.5; k=14) interventions, although the CIs overlapped substantially (Figure 2). None of the examined moderators reached significance in the omnibus Wald-type tests of the meta-regression models (QM): health domain (QM_5_=0.79, *P*=.56), delivery modality (QM_3_=1.22, *P*=.30), personal support (QM_1_=0.57, *P*=.45), incentives (QM_1_=1.03, *P*=.31), intervention duration (QM_1_=2.60, *P*=.11), or mean participant age (QM_1_=0.62, *P*=.43), with each moderator explaining essentially none of the between-study variance. An Egger-type regression test indicated possible small-study effects (z=−2.75, *P*=.007), with smaller studies tending to report lower attrition, although this test should be interpreted cautiously at this degree of heterogeneity (Figure 3).

**Figure 2.**
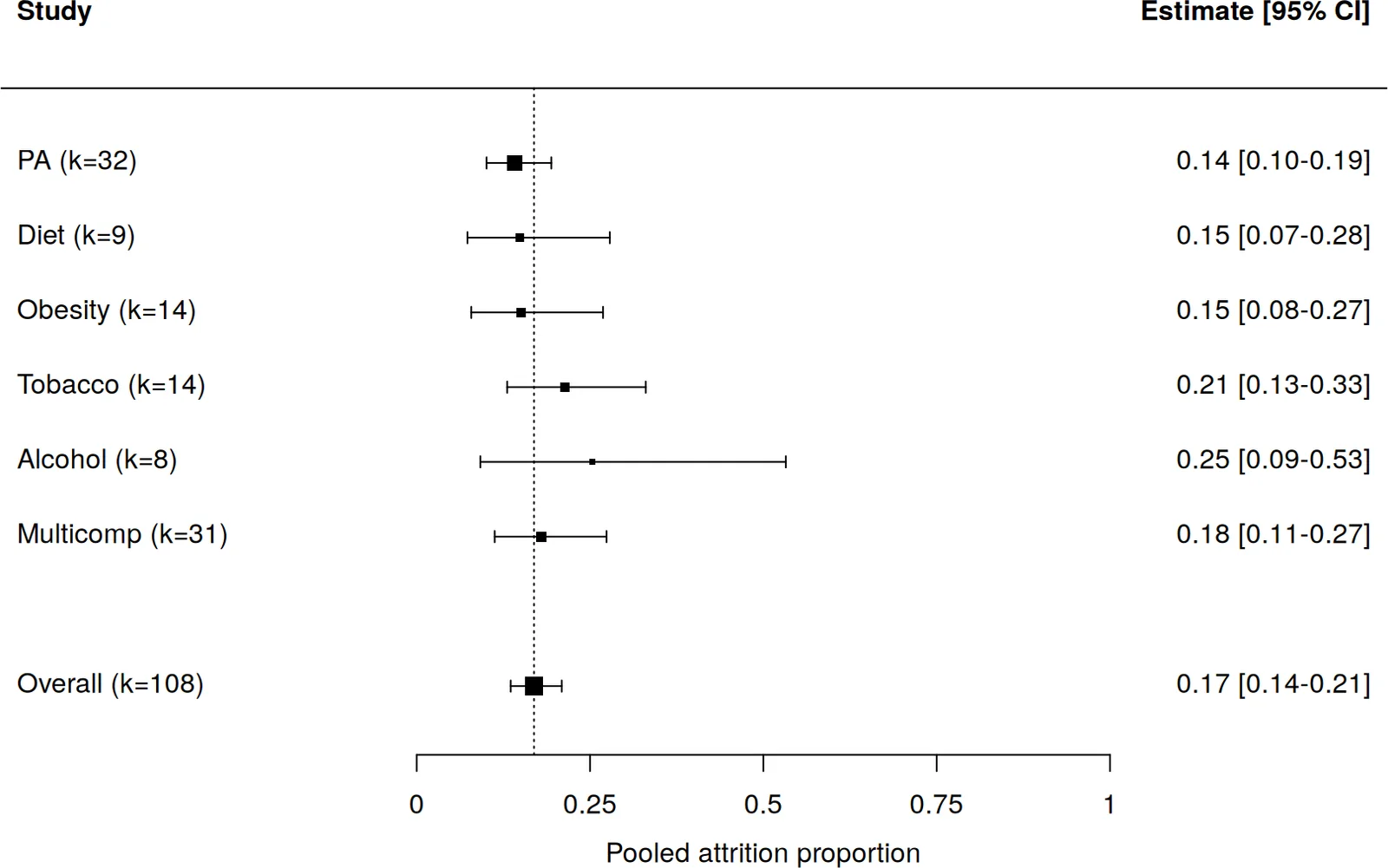
Forest plot of the random-effects meta-analysis of attrition. Pooled attrition 16.9% (95% CI 13.6‐20.9). PA: physical activity.

**Figure 3.**
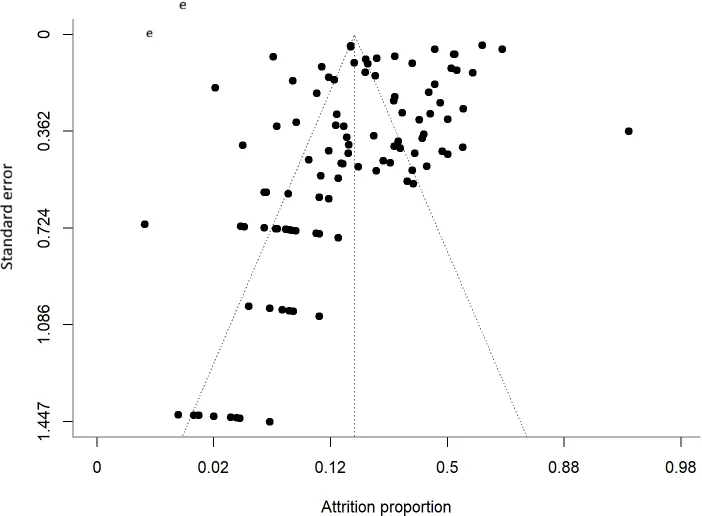
Funnel plot for the attrition meta-analysis (Egger-type test z=–2.75, *P*=.007).

Notably, pooled attrition (approximately 17%) was considerably lower than the degree of underuse implied by the completion and component-adherence metrics, under which roughly one-third of participants did not complete the full intervention, and approximately 45% of intervention components went unused. This pattern indicates that many adolescents remained nominally enrolled while substantially under-using the intervention, so that retention statistics alone overstate true adherence. Overall, attrition in adolescent digital lifestyle interventions is substantial but highly heterogeneous and is not explained by standard study-level characteristics.

### Physical Activity

#### Intervention-Related Factors

Several intervention design features were associated with adherence to physical activity interventions. Statistical evidence indicated higher adherence during daytime hours [20] and on weekdays versus weekends [21], while longer study duration was negatively associated with adherence [20,22]. Descriptive evidence reinforced the importance of specific program features, with activity trackers [23-25], outdoor and solitary usage [26], and targeted components including workout missions, storylines, parent-child activities, physical activity education, run logging, and week-runs [26] all showing positive associations with adherence. Further descriptive facilitators included the “Kids Zone” interface [24], coaching sessions [25], and coach reminder messages [21]. Conversely, descriptive evidence identified technical difficulties and equipment dissatisfaction [23,27], social intervention features [26], weekly challenges and Facebook videos (Meta Platforms, Inc) [28], poorly timed text messages during class time [28], educational modules [25], and excessive video duration [21] as barriers.

Qualitative evidence provided the richest insights into design-related facilitators and barriers. Participants consistently endorsed goal-setting components [28,29], tracking and monitoring features [26,28], flexible goal reminders [30], clear instructions with progressive difficulty scaling, encouragement, and immersive app design [26], and engaging activities including gamification elements and peer competition [27,28]. The provision of activity trackers (eg, Fitbits) was viewed positively [24], as were activity logging and local resource information [29], incentives [31], and diverse content including varied video and sports options [20,22]. However, qualitative data also revealed substantial technical barriers, including synchronization issues, device charging logistics, and website login difficulties [24,28,29,31,32], as well as equipment dissatisfaction due to perceived device inaccuracy [27,28,30]. Participants reported competition from alternative systems and information sources [24,28], unappealing website design with excessive text and insufficient youth-oriented content [24,29], limited social components [29], inadequate reward systems [31], and specific features such as message boards and expert forums that reduced adherence [29]. Participants expressed preferences for text messages over emails, which often went unread due to spam filtering [32].

The updated search added 10 physical activity studies that broadly reinforced these design-related patterns while adding nuance around support and gamification. Personalization again emerged as a facilitator, with tailored nurse feedback [77] and readiness-based content routing [78] supporting engagement, whereas a lack of customization lowered perceived usability [79]. Human support in its various forms was consistently valued, including nurse-led monitoring [77], weekly coaching calls [80], and live remote supervision [81], while the absence of therapist support was experienced as a barrier [78]. Gamification remained largely facilitative but was not uniformly positive, as competitive leaderboards discouraged lower-ranked users [82]. Technical and logistical friction continued to undermine adherence, with device charging and synchronization problems and simply forgetting to wear trackers reported repeatedly [83,84], whereas embedding the intervention in a familiar platform and delivering it remotely helped overcome access and geographic barriers [77,80].

#### User-Related Factors

User-related factors associated with adherence to physical activity interventions spanned demographic, motivational, and practical dimensions. Statistical evidence revealed that Hispanic ethnicity was positively associated with adherence [20]. Descriptive evidence identified gender-based motivational differences, with boys engaging primarily for enjoyment and girls participating to combat boredom [33], while injury, illness, and pain were associated with lower adherence [23]. Qualitative evidence highlighted that opportunities for family participation [28] and users’ perception of personal benefits [26] enhanced adherence, whereas lack of interest in the intervention [26,27], time constraints [23,26], and short-term motivation [28] acted as barriers. Practical challenges, including limited access to Wi-Fi or mobile data [28], insufficient device storage [32], and simply forgetting to engage with the intervention [31,32], were also reported as obstacles to sustained use.

The new physical activity studies extended the user-related picture, particularly for motivation, age, and clinical burden. Intrinsic motivation and perceived benefit again facilitated engagement, including satisfaction of competence and autonomy needs [85] and perceived health gains [78,80], whereas waning motivation and loss of interest drove disengagement [77,80,81]. The direction of age was not uniform, as older participants reported a higher intention to reuse a virtual exercise program than younger participants [86]. A new and distinct theme was illness burden in clinical samples, where fatigue, pain, comorbidities, hospitalizations, and fear of hypoglycemia limited participation [79-81]. Time constraints from school and competing demands, together with the poor fit of frequent device charging with busy routines, were again reported as obstacles [77,80,83].

### Obesity Management

#### Intervention-Related Factors

Several intervention design features were associated with adherence to digital obesity management interventions. Statistical evidence indicated that communication with a health coach [34,35], social voting polls [35], parental participation [35,36], online food journaling [37], reinforcement messages [38], primary recruitment sites [39], and in-class program delivery versus homework-based delivery [40] were significantly associated with increased adherence. Adherence was also significantly higher during the first half compared to the second half of interventions [36,41]. Descriptive evidence supported higher adherence rates associated with health coach communication [39,42], specific program features (My workouts, My steps, and My goal functions) [43], and the initial intervention phase [37,39,44]. Conversely, statistical evidence indicated that self-esteem and stress management message components [38] and the AppAlone program compared to the AppCoach program [39] were associated with reduced adherence. Descriptive evidence revealed barriers including the ineffective “my motivation” function [43] and the selected self-monitoring tool [36].

Qualitative evidence highlighted several facilitating features: informative videos covering topics such as nutrition label reading, heart rate measurement, and screen time health effects [45], daily encouragement with real-time feedback, gamification elements, peer competition, and accessible health care professional communication [46]. Users also positively perceived daily reminders, craving management strategies, and weekly coach phone calls [39]. However, qualitative evidence also revealed barriers including insufficient individualization and support, limited exercise variety, and inadequate in-person resources and group activities [47], technical difficulties [40,48], excessive and repetitive messaging [43], overly demanding training requirements regarding frequency and duration [48], and educator concerns about lesson length and reading volume [40].

#### User-Related Factors

User-related factors associated with adherence to obesity management interventions encompassed demographic, psychological, and behavioral dimensions. Statistical evidence demonstrated positive associations between adherence and successful BMI reduction in the initial 12 weeks [47], female gender [40], and younger age [40,44]. Additional significant predictors included intrinsic motivation, healthier parenting practices, and higher household income [44]. Conversely, statistical evidence indicated lower adherence associated with authoritative parenting style and depression [41,44]. Descriptive evidence revealed contrasting findings regarding socioeconomic status, with one study suggesting that lower household income was associated with better adherence [39]. Further descriptive evidence identified greater weight and shape concerns and white ethnicity [37], higher weight status, interfering activities, and scheduling conflicts such as being too busy, tired, or away from home [48] as barriers to adherence. Qualitative evidence revealed lost motivation as an additional barrier [48].

### Dietary Habits

#### Intervention-Related Factors

Several intervention design features were associated with adherence to dietary habit interventions. Statistical evidence indicated that text message frequency of 2‐3 messages per week was positively associated with adherence [49]. Descriptive evidence demonstrated positive effects from online resources and interactive quizzes [49], while a declining participation pattern was observed across the study duration [50]. Qualitative evidence supported the effectiveness of music integration, goal-setting functionalities, practical tips and recipes, food diary capabilities, and reminder systems [51,52], feedback mechanisms, device comfort, and visual design elements [52], as well as video content quality, language appropriateness, and overall layout structure [53]. However, qualitative evidence also revealed barriers including limited scenario applicability in dining hall settings and insufficient video content [53], inability to pause videos and excessive session duration [54], restricted reward options with specific requests for more song choices, and constrained food logging capabilities that only allowed tracking of fruits and vegetables [52].

Three new dietary studies reinforced the importance of tailoring, gamification, and content quality while highlighting reward-design pitfalls. Culturally and developmentally appropriate tailoring facilitated sustained use, including a slide-bar feature for shared family meals [87] and age-relevant rather than adult-oriented messaging [88]. Gamification elements such as badges, team goals, and social sharing were again linked to engagement [87], and credible, concise content was valued, although unmet requests for more recipes and features tempered enthusiasm [88]. Reward structures could backfire, however, as unattainable high-value rewards and delayed or embarrassing reward distribution discouraged engagement [89]. Technical and access constraints, including an Android-only app and limited school computer time, further limited use [89].

#### User-Related Factors

User-related factors associated with adherence to dietary interventions encompassed behavioral, psychological, and practical dimensions. Statistical evidence indicated positive associations between adherence and higher education levels, healthy snack consumption patterns, elevated self-efficacy scores for healthy eating, and perceived competence in app usage [50]. Descriptive evidence showed positive relationships with lower sensitivity to reward scores and maintenance of adherence among users experiencing positive emotional responses to the app [50]. Descriptive evidence also indicated that time constraints due to academic workload and general lack of available time hindered adherence [52].

The new dietary evidence on user characteristics was consistent with the previously established picture. Excessive or poorly understood notifications and overly long assessments were experienced as barriers that competed with adolescents’ limited time and attention [88]. Practical constraints on when and where the intervention could be used, such as restrictions on phone use at school, again shaped engagement [87,89].

### Tobacco Use

#### Intervention-Related Factors

Few intervention design features were identified in relation to adherence to nicotine use interventions. Descriptive evidence indicated that ease of use, engaging content, and the presence of a narrator enhanced adherence [55]. Statistical evidence demonstrated significant participant dropout on the designated “quit day” [56], and qualitative evidence identified technological implementation issues as barriers to adherence [57].

The updated search markedly strengthened the tobacco evidence, which had been sparse, adding 5 studies that clarified the roles of prompts, peer support, platform integration, and third-party technical dependencies. Multiple well-timed prompts significantly raised engagement among historically underengaged users [90], and integrating the intervention into clinical workflows or the electronic health record supported high overall engagement [90], as did opting participants in during an in-person visit rather than through later automated texts [91]. Peer-delivered support was a recurring facilitator, including counselor-facilitated group chats that raised perceived support [92] and peer-written messages that generated the highest engagement [93], although reactive rather than proactive peer coaching limited engagement [93]. Dependence on third-party platforms introduced new technical barriers, as mobile carriers automatically blocked a large share of outreach texts as spam [91] and changes to social media rules and handles drove attrition [92]. Low production quality and a single storyline also constrained engagement with a peer-video intervention [93].

#### User-Related Factors

User-related factors associated with adherence to nicotine use interventions were primarily identified through descriptive evidence. Facilitating characteristics included higher academic performance, regular school attendance, English as the home language, having internet access at home, and nonsmoking status [57]. Additional facilitators included extended prequit duration and multiple previous quit attempts (2 or more) [56]. Conversely, being a current or former smoker [57] and geographical location, specifically residing in the southern United States [56], were associated with lower adherence.

The new tobacco studies also enriched the user-related picture, which had previously rested on a single study, and introduced several within-domain contradictions. Higher baseline risk, including greater vaping frequency, dependence, and internalizing-symptom severity, predicted nonresponse [94], and non-susceptible never-smokers engaged more with prompts [90]. Motivation mattered, as low confidence to quit predicted nonresponse [94] and a quitting-centered framing did not resonate with adolescents who did not identify as smokers [91]. Gender associations pointed in opposite directions across studies, with male gender predicting nonresponse in one program [94] but female gender associated with greater attrition in another [92]. A distinctively adolescent barrier was privacy, as participants were reluctant to text a clinician while at school or near a parent, and home delivery of nicotine replacement risked forcing disclosure to parents [91].

### Alcohol Use

#### Intervention-Related Factors

Few intervention design features were identified in relation to adherence to alcohol consumption interventions. Descriptive evidence supported that message frequency, particularly 1‐3 messages per week, was associated with higher adherence [58]. Conversely, descriptive evidence showed that home-based booster sessions, compared to school settings, were associated with lower adherence [59]. Qualitative evidence revealed technical barriers impacting adherence, including ineffective email delivery resulting in messages being directed to spam folders and general logistical difficulties [59].

Three newly identified alcohol studies reinforced the roles of usability, tailoring, and prompts while adding contradicting evidence on self-monitoring. A positive overall evaluation of an eHealth game predicted higher usability ratings, and its attractive layout and gamified avatars, rewards, and stories were well received [95], while tailored feedback supported engagement in a web-based program [96]. A peer-to-peer nudge strongly predicted program completion [97]. Counterbalancing the facilitative view of self-monitoring, one program found no dose-response between content exposure and outcomes [96]. Over-long or repetitive elements, including film lectures and baseline questionnaires, and a logout-and-break structure that discouraged reengagement, were reported as barriers [95,96].

#### User-Related Factors

User-related factors associated with adherence to alcohol consumption interventions spanned demographic and behavioral dimensions. Statistical evidence demonstrated higher adherence among females, younger individuals, and those with higher educational levels [60], as well as participants of Spanish nationality [59]. Descriptive evidence indicated additional facilitating characteristics, including younger age [58,59], Dutch nationality [60], female gender, and higher maximum drinks per occasion [58]. Furthermore, descriptive evidence suggested that lower weekly alcohol consumption was linked to better adherence [59,60]. Conversely, descriptive evidence identified barriers including commencement of workplace placements during the intervention period, logistical challenges related to school commitments, and lower educational attainment [59].

The newly identified alcohol studies broadened the user-related determinants, especially around baseline risk, family factors, and socioeconomic status. Contrary to the prior pattern, adolescents reporting binge drinking rated the program more positively and engaged more with its action-planning content [95], while depression was linked to low adherence in a highly distressed sample [96]. Family and socioeconomic factors were prominent, as parental communication self-efficacy and open communication predicted completion and retention, higher household income raised initiation, and full-time parental employment aided retention [97]. Age and gender associations were mixed, with younger teens showing greater retention and completion [97] but older adolescents more likely to recommend the program [95]. Notably, self-identification with an ethnic category other than White was associated with greater retention [97], a facilitator direction that contrasts with the barrier role of minority status seen in the tobacco domain.

### Multicomponent Lifestyle Interventions

#### Intervention-Related Factors

Several intervention design features were associated with adherence to digital multicomponent lifestyle interventions. Statistical evidence indicated that high parent engagement [61], self-regulation and narrative components [62], and reminder messages [63] were significantly associated with increased adherence. Descriptive evidence highlighted several facilitating factors: comfortable equipment [64], easy handling [65], incentives, access to new information, and progress feedback [66]. Additional positive effects were observed from point systems [67], quizzes [68,69], recipe provision [68], SMS prompts, prizes, and 4-month program duration [69,70]. The social skills component showed high participation rates [69,70], with peak interaction times observed between 3 PM and 1 AM on weekdays and weekends [71]. Conversely, statistical evidence demonstrated reduced adherence associated with smoking cessation stage-of-change assessments and contest prompts [72], as well as picture and message contests [69,70]. Descriptive evidence identified barriers including uncomfortable heart rate monitors [64], limited new website content [66], and declining participation rates over extended study durations [61,68,71].

Qualitative evidence supported the effectiveness of a concise baseline questionnaire and platform design [73], friendly health competitions and small altruistic rewards [74], and motivational support features [75]. However, qualitative evidence also revealed challenges related to poorly timed surveys [64], time-intensive baseline questionnaires, and technical difficulties encountered during the intervention [75].

Twelve newly identified multicomponent studies substantially expanded this domain and, in several cases, complicated the earlier facilitator picture. Gamification and reward mechanics were again linked to engagement, including health coins, streaks, and unlockable content associated with high weekly logins [98] and smartwatch reward displays that motivated use [99], whereas the absence of gamification was experienced as a barrier [106]. The clearest complication concerned self-monitoring: although tracking with feedback facilitated engagement in some programs [99,100], the perceived burden of daily self-monitoring reduced adherence and conferred no advantage over paper records in one trial [101], and self-monitoring components saw low or only partial uptake elsewhere [102,103,110]. Parental involvement remained facilitative, with caregiver-adolescent engagement correlated [104] and a mother a more acceptable co-participant than a friend [105]. Engagement typically declined over time, with effects lost by 18 months and most participants stopping after a few weeks [105,106], while shorter duration and completion incentives reduced dropout [107]. Incentives showed mixed effects, raising short-term motivation that faded on withdrawal [104] and, when coercively grade-linked, degrading app perception with heavier use [108].

#### User-Related Factors

User-related factors associated with adherence to multicomponent lifestyle interventions were extensive. Statistical evidence showed higher adherence among females, individuals with weaker intentions to consume fruits and vegetables, and British nationals [73], as well as those with less exercise at baseline, lower BMI, and better health status [75]. Additional statistical predictors included mothers versus fathers in parent-child dyads and parents with high education [61], younger participants [76], those who had been drunk in the past month, and previous users of the internet for health purposes [63], as well as individuals with higher perceived benefits of quitting [72]. Descriptive evidence indicated increased adherence among males [74], individuals with higher risk perception of drugs, lower alcohol and tobacco consumption, and fewer family-related alcohol and drug abuse problems [63], as well as younger participants, females, and those with nonproblematic drinking patterns [69,70]. Qualitative evidence highlighted enhanced adherence when participants felt part of a larger initiative and enjoyed learning about health and engaging in personal challenges [64].

Conversely, statistical evidence identified lower adherence among adolescents from nonacademic educational tracks compared to academic tracks [62], those with religious affiliation [63], and individuals with older age, binge drinking behavior, and immigration background [72]. Descriptive evidence revealed barriers including time constraints [66], perceived lack of behavioral change when using the app, subjective perception of already maintaining a healthy lifestyle, and insufficient environmental motivation [62], concurrent use of alternative lifestyle tracking apps and channels [62,63], lost login credentials [68], lack of interest [63,68], engagement in extracurricular activities, school absences, relationship status, and preference for alternative communication channels [63], and immigration background [69,70]. Qualitative evidence highlighted additional barriers including time limitations [74], insufficient device storage capacity, and the perception of mandatory participation [62].

The newly identified multicomponent studies also enriched the user-related determinants and reinforced several barriers. Lower socioeconomic position was again implicated, as noncompleters had significantly lower paternal education [101]. Loss of interest and waning motivation were among the most common reasons for dropout [98,101,106], and competing demands on adolescents’ time, including school examinations, travel distance, and parental work, repeatedly limited engagement [99,101,103,110]. Access and device constraints, including device dependency and exclusion of adolescents without smartphones, raised digital-divide concerns [98,99]. A recurring adolescent-specific theme was privacy, which cut in both directions: a private, user-controlled interface reduced fear of judgment [101], yet a program that felt too private discouraged involving a friend and some adolescents preferred passive, nonsocial participation [102,105]. A late-responder subgroup with a mixed risk profile further illustrated that engagement timing varies across adolescents [109].

Twenty-six of the included studies contributed only to the descriptive synthesis of study characteristics and, where attrition data were extractable, to the quantitative synthesis of attrition; no factors influencing adherence were reported in these studies, and they therefore do not appear in the factor-level synthesis presented in Tables S1-S6 Multimedia Appendix 1. They comprised studies of physical activity [119-125], dietary habits [126], obesity management [127-130], tobacco use [131-136], alcohol use [137-140], multicomponent lifestyle interventions [141-143], and the study by Parker et al [147].

## Discussion

### Principal Findings

This systematic review synthesized evidence from 116 studies to identify the factors influencing adherence to digital lifestyle interventions for adolescents. Our analysis revealed that, although just over half (47/89, 52.8%) of the interventions successfully achieved their primary behavioral outcomes, this effectiveness is likely to be undermined by significant adherence challenges. On average, participants completed 66.9% (SD 26%) of the interventions (the mean completion rate across the 68 comparisons that reported it). Mean adherence to individual intervention components was lower still: 55.2% (SD 25.5%) (across the 66 comparisons that reported it). This disparity highlights a critical issue, namely that an intervention is unlikely to be effective if users do not engage with it as intended [148]. Therefore, understanding the drivers of adherence is crucial.

### Intervention-Related Determinants of Adherence

Several intervention-related elements were consistently effective in promoting adherence across multiple health domains. Personalization capabilities, intuitive user interfaces, strategically timed reminder systems, goal-setting functionalities, and gamification elements were found to be particularly influential design features. These findings align with and extend research on adult populations. For instance, the importance of personalization and user-friendly design echoes findings from systematic reviews on digital mental health interventions for adults [10]. However, our review indicates that for adolescents, these elements may need to be combined with gamification and engaging activities to be successful. This is consistent with self-determination theory, which posits that supporting autonomy (personalization), competence (usability and goal-setting), and relatedness (human support) fosters the intrinsic motivation crucial for sustained engagement [5,149]. The effectiveness of gamification elements, such as music integration, interactive quizzes, and competitive features, suggests that interventions that leverage intrinsic motivation through enjoyable activities may achieve better adherence outcomes than purely informational approaches [150]. Importantly, while gamification and “engaging activities” are related, they are operationally distinct in the included studies. Gamification refers to the application of game-design elements (eg, points, badges, leaderboards, and competition) to nongame contexts [151], whereas engaging activities encompass a broader category of design features intended to sustain interest, including interactive quizzes, music integration, narrative storylines, and varied content formats. Both contributed to adherence, but through different mechanisms: gamification primarily leverages extrinsic motivational pathways through reward and competition, while engaging activities may support intrinsic engagement through enjoyment and curiosity.

The prominent role of human support elements, which is particularly evident in obesity interventions through health coach communication and parental involvement, highlights the ongoing importance of interpersonal connections in digital health contexts [5,152]. This finding supports the emphasis placed on social support as a critical determinant of behavior change maintenance in social cognitive theory [153,154]. However, the mixed evidence regarding the effectiveness of social intervention components, such as message boards and expert forums, suggests that not all forms of social interaction enhance adherence. The effectiveness of human support may depend on the quality, timing, and perceived relevance of the interaction, rather than on the mere availability of social features.

Implementing comprehensive human support presents significant scalability challenges, particularly for universal prevention programs, where providing individual attention becomes logistically and economically unfeasible. Constraints on resources in health care and education systems often prevent the provision of sustained professional support for large groups of participants. While hybrid models combining digital tools with personal support can optimize adherence in clinical or targeted prevention contexts, alternative strategies deserve consideration for population-level implementations. These could include peer mentoring systems, AI-driven personalized feedback mechanisms, or tiered support models where human interaction is strategically deployed for participants showing signs of early disengagement or elevated risk [155].

Technical implementation barriers consistently emerged as substantial obstacles to sustained adherence in all health domains, regardless of the focus of the specific intervention. Synchronization difficulties, authentication problems, and inappropriately timed notifications emphasize the importance of robust technological infrastructure and comprehensive, user-centered design principles. These technical challenges were particularly evident in interventions targeting physical activity and alcohol use, potentially due to the real-time tracking requirements and environmental context sensitivity inherent to these health domains. The consistent finding of declining adherence over the duration of interventions [156], particularly in dietary and multicomponent domains, suggests the necessity of adaptive engagement strategies that evolve dynamically to maintain user interest and combat habituation effects.

### User-Related Determinants of Adherence

The synthesis revealed complex and occasionally contradictory relationships between demographic characteristics and adherence patterns. This indicates that associations are context-dependent and cannot be easily generalized. While some studies identified higher adherence among female participants and younger adolescents, these relationships demonstrated considerable variability across health domains and study contexts. This suggests that demographic predictors may be moderated by intervention characteristics, the health domain in question, and cultural factors. This heterogeneity is consistent with ecological systems theory, which emphasizes the multifaceted influences on adolescent behavior, including individual, family, peer, and broader societal factors [157].

Socioeconomic indicators, particularly educational attainment and household income, exhibited more consistent associations with adherence; however, the nature of these relationships varied across health domains. Higher educational levels were associated with better adherence to dietary and alcohol interventions, which may reflect enhanced health literacy and digital competency. However, conflicting evidence regarding the effects of household income on obesity interventions was found, suggesting that socioeconomic influences may operate differently across behavioral targets. These findings emphasize the importance of considering the implications for health equity in the design of digital interventions and the potential need for differentiated implementation strategies across socioeconomic groups.

These socioeconomic patterns in adherence have important implications for health equity that extend beyond the adolescent-specific literature examined here. Systematic reviews examining digital health interventions in adult populations have identified similar socioeconomic gradients in engagement. Szinay et al [158] found that the uptake, engagement with, and effectiveness of mobile interventions for weight-related behaviors were not equally distributed across socioeconomic groups, raising concerns that digital health interventions may inadvertently widen health inequalities. Western et al [159] further argued that the “digital health divide” operates at multiple levels, including individual digital literacy, infrastructure access, and the socioeconomic context of health domains. Our findings in adolescent populations echo these adult-focused reviews and suggest that the equity implications of digital health interventions may emerge early in the lifespan. Interventions targeting adolescents from lower socioeconomic backgrounds may require differentiated design and implementation strategies, such as offline functionality, reduced data requirements, or integration with existing community infrastructure, to ensure equitable engagement.

Psychological factors, particularly intrinsic motivation and the perceived personal benefits of the intervention, emerged as powerful and consistent facilitators. This is particularly relevant for adolescents, who, as noted in the literature, are in a critical period of identity formation during which their cognitive decision-making and critical thinking functions are still maturing [11,12]. Interventions that do not align with their developing personal values or are not perceived as immediately relevant or beneficial are likely to be abandoned, which is consistent with motivation theories that emphasize the need for perceived efficacy and value alignment to encourage long-term participation [149,160]. However, the frequent reporting of loss of motivation and declining interest as barriers to adherence, particularly in the obesity and physical activity domains, highlights the difficulty of maintaining initial enthusiasm throughout extended intervention periods.

### Domain-Specific Adherence Patterns

Adherence-related outcomes varied across health domains, illuminating the different challenges and motivators associated with specific behavioral targets. In the quantitative synthesis of attrition, dropout was lowest in physical activity interventions, potentially reflecting the immediate feedback and tangible outcomes associated with movement tracking, and highest in alcohol and tobacco interventions, possibly reflecting the sensitive nature of substance-use disclosure and the complex psychological factors surrounding these behaviors in adolescents. Completion and component-adherence rates also differed across domains, although the wide and overlapping ranges caution against overinterpreting any single domain ordering.

Multicomponent lifestyle interventions illustrate the tension between comprehensiveness and adherence. While targeting multiple risk factors simultaneously is theoretically appealing, the expanded evidence base indicated that such programs frequently encountered engagement decline, self-monitoring burden, and competing demands, suggesting that they may present unique adherence challenges that require specialized design considerations to maintain participant involvement across diverse behavioral targets.

Quantitatively synthesizing attrition reinforced and sharpened the narrative findings. Although a substantial minority of adolescents (pooled 16.9%) dropped out of the included studies, the between-study heterogeneity was extreme (*I*^2^>98%) and was not explained by health domain, delivery modality, human support, incentives, intervention duration, or participant age. The level of disengagement is highly context-specific and is not predicted by the broad design characteristics that are typically reported, which supports our central recommendation that the field should adopt standardized adherence metrics. The contrast between modest attrition (approximately 17%) and lower intervention use, with mean completion of approximately two-thirds and mean component adherence of approximately one-half, is equally instructive. Adolescents tend to remain nominally enrolled while ceasing to engage with the intervention as intended, so that retention statistics alone substantially overstate true adherence.

These quantitative patterns are consistent with recent syntheses reporting low and declining real-world engagement with digital interventions among young people and a comparable facilitator and barrier structure [161-164].

### Limitations

This systematic review has several important limitations. First, the study protocol was not prospectively registered, and we did not conduct forward or backward citation searching, which may have resulted in the omission of relevant studies not indexed in the searched databases. Second, we did not conduct formal risk of bias assessments of included studies. While validated tools exist for assessing risk of bias across different study designs (eg, the revised Cochrane Risk of Bias tool for randomized trials [RoB 2] [165] and the Risk Of Bias In Non-randomized Studies of Interventions [ROBINS-I] tool [166]), our primary objective was to identify and categorize factors reported as influencing adherence rather than to evaluate intervention effectiveness. Because we do not aggregate effect estimates or draw causal conclusions about intervention efficacy, a formal risk-of-bias assessment of each study’s primary outcomes would not directly inform our narrative synthesis. We acknowledge, however, that this means findings from studies with varying methodological rigor receive equal weight in our synthesis. Third, this review focused on 5 lifestyle-related health domains selected based on their relevance to cancer prevention. The findings may not be generalizable to digital interventions targeting other health domains, such as mental health or sexual health, where the developmental and behavioral contexts differ substantially. Finally, although attrition could be meta-analyzed, the pooled estimates should be interpreted in light of very high between-study heterogeneity and the inconsistent operationalization of attrition and adherence across studies; the estimates are descriptive, are not weighted by study-level risk of bias, and the small-study-effect test should be read cautiously at this level of heterogeneity. Completion and component-level adherence were reported too heterogeneously to support a defensible pooled estimate and were therefore synthesized descriptively and through the SWiM approach rather than meta-analytically.

### Implications for Future Research

The findings of this review have several implications for future research and practice. First, the considerable heterogeneity in adherence measurement and reporting represents a fundamental barrier to advancing the field. We recommend that future studies adopt standardized adherence metrics, distinguishing at minimum between completion rates (proportion completing the intervention) and component-level adherence (engagement with specific intervention elements), and report both measures alongside their operationalization. Second, the consistent role of intervention design features across multiple health domains suggests that these elements should be considered core components in the design of digital health interventions for adolescents. Third, the mixed evidence regarding social features highlights the need for experimental research that systematically varies the type, timing, and intensity of social components to determine optimal configurations for different target groups. Fourth, the clear impact of technical barriers across all domains underscores the importance of rigorous usability testing and iterative design processes in intervention development. Finally, future research should explicitly examine the dose-response relationship between adherence and intervention effectiveness. Meta-analyses of individual participant data could provide enhanced statistical power for identifying optimal intervention parameters while accounting for participant-level moderators of adherence [167].

### Conclusion

This systematic review provides a comprehensive overview of the factors influencing adherence to digital lifestyle interventions among adolescents. The findings show that intervention-related design features and user-related characteristics consistently affect adherence patterns, although these relationships are often context- and domain-specific. Complementing this narrative synthesis, a random-effects meta-analysis of attrition across 108 comparisons produced a pooled dropout of approximately 17%, but with extreme between-study heterogeneity that none of the examined study-level moderators explained, reinforcing that the degree of disengagement is highly context-specific rather than predictable from broad design features. The substantial variation in adherence rates and measurement approaches highlights the urgent need to establish standardized reporting frameworks that incorporate validated measures and clearly defined usage parameters. Such methodological advancements are essential for synthesizing robust evidence and translating research findings into effective, engaging digital health interventions for adolescent populations.

## Supplementary material

10.2196/84822 Multimedia Appendix 1 Search strategies, factors influencing adherence by health domain (Tables S1-S6), characteristics and adherence-related characteristics of the included studies (Tables S7-S18), and the Synthesis Without Meta-analysis effect-direction table.

10.2196/84822 Checklist 1 PRISMA 2020 checklist.

## Abbreviations

PEO: Population-Exposure-Outcome
PRISMA: Preferred Reporting Items for Systematic Reviews and Meta-Analyses
RCT: randomized controlled trial
RoB 2: Cochrane Risk of Bias tool for randomized trials
ROBINS-I: Risk Of Bias In Non-randomized Studies of Interventions
SUNRISE: Sustainable Interventions and Healthy Behaviours for Adolescent Primary Prevention of Cancer with Digital Tools
SWiM: Synthesis Without Meta-analysis
WHO: World Health Organization
