# Supplementary material for: Factors Influencing Adherence to Digital Lifestyle Interventions for Adolescents: Systematic Review and Meta-Analysis of Attrition
Source: Interact J Med Res. 2026 Sep 30;15:e84822. doi: 10.2196/84822 (PMC13626193; doi:10.2196/84822)
Supplement: Supplementary file 2 [file ijmr-v15-e84822-s001.docx]

**Appendix S1.**

The searches reported in this appendix were first conducted in March 2024 and re-run without modification on 22 June 2026 to update the review. Identical search strings were used in both search waves across all four databases.

**PubMed search string.**

“Internet”[Mesh] OR “Mobile applications”[Mesh] OR “Computers”[Mesh] OR “Therapy, Computer-Assisted”[Mesh] OR “Telemedicine”[Mesh] OR “Mobile Applications”[Mesh] OR “Internet-Based Intervention”[Mesh] OR “Internet”[All Fields] OR “Mobile applications”[All Fields] OR “Computer”[All Fields] OR “Computer-assisted”[All Fields] OR “Telemedicine”[All Fields] OR “Mobile Applications”[All Fields] OR “Internet-Based Intervention”[All Fields] OR “Online”[All Fields] OR “Web”[All Fields] OR “E-health”[All Fields] OR “Mobile phone”[All Fields] OR “Smartphone”[All Fields] OR “Mobile device”[All Fields] OR “Tablet”[All Fields] OR “App”[All Fields] OR “CD-ROM”[All Fields] OR “digital health”[All Fields] OR “eHealth”[All Fields] OR “digital intervention”[All Fields] OR “online program”[All Fields] OR “web-based”[All Fields] OR “app-based”[All Fields] OR “mobile health”[All Fields] OR “SMS”[All Fields] OR “text messag*”[All Fields] OR “messenger”[All Fields]

“Patient Compliance”[Mesh] OR “Treatment Adherence and Compliance”[Mesh] OR “Patient Dropouts”[Mesh] OR “adherence”[All Fields] OR “compliance”[All Fields] OR “engagement”[All Fields] OR “participation”[All Fields] OR “dropout”[All Fields] OR “retention”[All Fields] OR “engag*”[All Fields] OR “adhere*”[All Fields] OR “retain*”[All Fields] OR “drop-out”[All Fields] OR “disengag*”[All Fields] OR “usage”[All Fields] OR “intended use”[All Fields] OR “non-adhere*”[All Fields] OR “nonadhere*”[All Fields] OR “non-compli*”[All Fields] OR “noncompli*”[All Fields] OR “program use”[All Fields] OR “intervention use”[All Fields]

"smoking"[All Fields] OR "nicotine"[All Fields] OR "tobacco use"[All Fields] OR "smoking cessation"[All Fields] OR "cigarette smoking"[All Fields] OR "vaping"[All Fields] OR "e-cigarettes"[All Fields] OR "tobacco cessation"[All Fields] OR "nicotine dependence"[All Fields] OR "Smoking"[MeSH] OR "Tobacco Use Disorder"[MeSH] OR "Smoking Cessation"[MeSH] OR "Nicotine"[MeSH] OR "obesity"[All Fields] OR "overweight"[All Fields] OR "weight management"[All Fields] OR "body weight"[All Fields] OR "body mass index"[All Fields] OR "BMI"[All Fields] OR "obese"[All Fields] OR "weight reduction"[All Fields] OR "weight control"[All Fields] OR "Obesity"[MeSH] OR "Body Weight"[MeSH] OR "Overweight"[MeSH] OR "Weight Loss"[MeSH] OR "diet"[All Fields] OR "dietary habits"[All Fields] OR "nutrition"[All Fields] OR "nutritional intervention"[All Fields] OR "eating habits"[All Fields] OR "food"[All Fields] OR "diet therapy"[All Fields] OR "dietary patterns"[All Fields] OR "healthy diet"[All Fields] OR "Diet"[MeSH] OR "Diet Therapy"[MeSH] OR "Feeding Behavior"[MeSH] OR "Nutritional Status"[MeSH] OR "alcohol"[All Fields] OR "drinking"[All Fields] OR "alcohol consumption"[All Fields] OR "alcoholism"[All Fields] OR "alcoholic beverages"[All Fields] OR "binge drinking"[All Fields] OR "alcohol abuse"[All Fields] OR "alcohol use disorder" OR "Alcohol Drinking"[MeSH] OR "Alcoholism"[MeSH] OR "Alcoholic Beverages"[MeSH] OR "Binge Drinking"[MeSH] OR "physical activity"[All Fields] OR "exercise"[All Fields] OR "fitness program"[All Fields] OR "physical fitness"[All Fields] OR "aerobic exercise"[All Fields] OR "sports"[All Fields] OR "physical exertion"[All Fields] OR "sedentary lifestyle"[All Fields] OR "active lifestyle"[All Fields] OR "Exercise"[MeSH] OR "Physical Fitness"[MeSH] OR "Physical Exertion"[MeSH]"

**Embase search string.**

'Internet'/exp OR 'mobile application'/exp OR 'computer'/exp OR 'computer assisted therapy'/exp OR 'telemedicine'/exp OR 'mobile application'/exp OR 'web-based intervention'/exp OR 'internet' OR 'mobile applications' OR 'computer' OR 'computer-assisted' OR 'telemedicine' OR 'mobile applications' OR 'internet-based intervention' OR 'online' OR 'web' OR 'e-health' OR 'mobile phone' OR 'smartphone' OR 'mobile device' OR 'tablet' OR 'app' OR 'cd-rom' OR 'digital health' OR 'ehealth' OR 'digital intervention' OR 'online program' OR 'web-based' OR 'app-based' OR 'mobile health' OR 'sms' OR 'text messag*' OR 'messenger'

'patient compliance'/exp OR 'treatment adherence' OR 'compliance (physical)'/exp OR 'patient dropout'/exp OR 'adherence' OR 'compliance' OR 'engagement' OR 'participation' OR 'dropout' OR 'retention' OR 'engag*' OR 'adhere*' OR 'retain*' OR 'drop-out' OR 'disengag*' OR 'usage' OR 'intended use' OR 'non-adhere*' OR 'nonadhere*' OR 'non-compli*' OR 'noncompli*' OR 'program use' OR 'intervention use'

'smoking' OR 'nicotine' OR 'tobacco use' OR 'smoking cessation' OR 'cigarette smoking' OR 'vaping' OR 'e-cigarettes' OR 'tobacco cessation' OR 'nicotine dependence' OR 'smoking'/exp OR 'tobacco dependence'/exp OR 'smoking cessation'/exp OR 'nicotine'/exp OR 'obesity' OR 'overweight' OR 'weight management' OR 'body weight' OR 'body mass index' OR 'bmi' OR 'obese' OR 'weight reduction' OR 'weight control' OR 'obesity'/exp OR 'body weight'/exp OR 'obesity'/exp OR 'body weight loss'/exp OR 'diet' OR 'dietary habits' OR 'nutrition' OR 'nutritional intervention' OR 'eating habits' OR 'food' OR 'diet therapy' OR 'dietary patterns' OR 'healthy diet' OR 'diet'/exp OR 'diet therapy'/exp OR 'feeding behavior'/exp OR 'nutritional status'/exp OR 'alcohol' OR 'drinking' OR 'alcohol consumption' OR 'alcoholism' OR 'alcoholic beverages' OR 'binge drinking' OR 'alcohol abuse' OR 'alcohol use disorder' OR 'drinking behavior'/exp OR 'alcoholism'/exp OR 'alcoholic beverage'/exp OR 'binge drinking'/exp OR 'physical activity' OR 'exercise' OR 'fitness program' OR 'physical fitness' OR 'aerobic exercise' OR 'sports' OR 'physical exertion' OR 'sedentary lifestyle' OR 'active lifestyle' OR 'exercise'/exp OR 'fitness'/exp OR 'exercise'/exp OR 'sedentary lifestyle'/exp

**PsycInfo search string.**

DE "Internet" OR DE "Mobile Applications" OR DE "Computer Applications" OR DE "Computer Assisted Therapy" OR DE "Telemedicine" OR DE "Digital Interventions" OR “internet” OR "Mobile applications" OR “computer” OR "Computer-assisted" OR “telemedicine” OR "Mobile Applications" OR "Internet-Based Intervention" OR “online” OR “web” OR "E-health" OR "Mobile phone" OR “smartphone” OR "Mobile device" OR “tablet” OR “app” OR "CD-ROM" OR "digital health" OR “eHealth” OR "digital intervention" OR "online program" OR “web-based” OR “app-based” OR "mobile health" OR “SMS” OR “text messag*” OR “messenger”

DE "Treatment Dropouts" OR DE "Treatment Compliance" OR “adherence” OR “compliance” OR “engagement” OR “participation” OR “dropout” OR “retention” OR “engag*” OR “adhere*” OR "retain*" OR "drop-out" OR “disengag*” OR “usage” OR "intended use" OR "non-adhere*" OR "nonadhere*" OR "non-compli*" OR "noncompli*" OR “program use” OR “intervention use”

“smoking” OR “nicotine” OR "tobacco use" OR "smoking cessation" OR "cigarette smoking" OR “vaping” OR "e-cigarettes" OR "tobacco cessation" OR "nicotine dependence" OR DE "Smoking Prevention" OR DE "Tobacco Smoking" OR DE "Smoking Cessation" OR DE "Nicotine" OR “obesity” OR “overweight” OR "weight management" OR "body weight" OR "body mass index" OR “BMI” OR “obese” OR "weight reduction" OR "weight control" OR DE "Obesity" OR DE "Body Weight" OR DE "Overweight" OR DE "Weight Loss" OR “diet” OR "dietary habits" OR “nutrition” OR "nutritional intervention" OR "eating habits" OR "food" OR "diet therapy" OR "dietary patterns" OR "healthy diet" OR DE "Diets" OR DE "Dietary Restraint" OR DE "Dietary Treatment" OR “alcohol” OR “drinking” OR "alcohol consumption" OR “alcoholism” OR "alcoholic beverages" OR "binge drinking" OR "alcohol abuse" OR "alcohol use disorder" OR DE "Alcohol Intoxication" OR DE “Alcohol treatment” OR DE "Alcoholism" OR DE "Alcohol Abuse" OR DE "Alcohol Use" OR DE "Alcoholic Beverages" OR DE “Alcohol Use Attitudes” OR "physical activity" OR “exercise” OR "fitness program" OR "physical fitness" OR "aerobic exercise" OR “sports” OR "physical exertion" OR "sedentary lifestyle" OR "active lifestyle" OR DE "Exercise" OR DE "Physical Fitness" OR DE "Physical Activity" OR DE "Sedentary Behavior"

**CINAHL search string.**

MH "Internet" OR MH "Mobile Applications" OR MH "Computers and Computerization" OR MH "Therapy, Computer Assisted" OR MH "Telemedicine" OR MH "Mobile Applications" OR MH "Internet-Based Intervention" OR “Internet” OR "Mobile applications" OR “Computer” OR "Computer-assisted" OR “Telemedicine” OR "Mobile Applications" OR "Internet-Based Intervention" OR “Online” OR “Web” OR "E-health" OR "Mobile phone" OR “Smartphone” OR "Mobile device" OR “Tablet” OR “App” OR "CD-ROM" OR "digital health" OR “eHealth” OR "digital intervention" OR "online program" OR “web-based” OR “app-based” OR "mobile health" OR SMS OR "text messag*" OR “messenger”

MH "Patient Compliance" OR MH "Treatment Refusal" OR MH "Patient Dropouts" OR MH “Research Dropouts” OR “adherence” OR “compliance” OR “engagement” OR “participation” OR “dropout” OR “retention” OR “engag*” OR “adhere*” OR "retain*" OR "drop-out" OR “disengag*” OR “usage” OR "intended use" OR "non-adhere*" OR "nonadhere*" OR "non-compli*" OR "noncompli*" OR "program use" OR "intervention use"

“smoking” OR “nicotine” OR "tobacco use" OR "smoking cessation" OR "cigarette smoking" OR “vaping” OR "e-cigarettes" OR "tobacco cessation" OR "nicotine dependence" OR MH "Smoking" OR MH "Tobacco" OR MH "Smoking Cessation" OR MH "Nicotine" OR “obesity” OR “overweight” OR "weight management" OR "body weight" OR "body mass index" OR BMI OR “obese” OR "weight reduction" OR "weight control" OR MH "Obesity" OR MH "Body Weight" OR MH "Nutrition" OR MH "Weight Loss" OR “diet” OR "dietary habits" OR “nutrition” OR "nutritional intervention" OR "eating habits" OR “food” OR "diet therapy" OR "dietary patterns" OR "healthy diet" OR MH "Diet" OR MH "Diet Therapy" OR MH "Eating Behavior" OR MH "Nutritional Status" OR “alcohol” OR “drinking” OR "alcohol consumption" OR “alcoholism” OR "alcoholic beverages" OR "binge drinking" OR "alcohol abuse" OR "alcohol use disorder" OR MH "Alcohol Drinking" OR MH "Alcoholism" OR MH "Alcoholic Beverages" OR MH “Alcohol Abuse” OR MH "Binge Drinking" OR "physical activity" OR “exercise” OR "fitness program" OR "physical fitness" OR "aerobic exercise" OR “sports” OR "physical exertion" OR "sedentary lifestyle" OR "active lifestyle" OR MH "Exercise" OR MH "Physical Fitness" OR MH "Life Style, Sedentary"

**Appendix S2**

**Table S1. Factors influencing adherence in the health domain of physical activity.**

| Intervention-related Factors | User-related Factors |
| --- | --- |
| **Facilitators**  Statistically^a^   - Increased compliance across the day[20][20] - Weekdays compared to weekends [21]   Descriptive*^b^*   - Workout mission tasks [26] - Storyline [26] - Run log and week-runs [26] - Use outdoors and while alone [26] - Activities with parents and learning about physical activity [26] - Activity tracker [23–25] - “Kids Zone” page [24] - Coaching sessions [25] - Reminder messages from coaches [21] - Adequate workout difficulty level [86] - Ease of use and willingness to recommend [83] - High device-wear and acceptability among completers [84] - Academic-credit incentive for completion [85] - Gamification (experience points, ranking) [79] [77] - High ease of use [79] - Parental involvement via shared dashboard [82] - Integration within a familiar platform (WeChat) [77] - Nurse-led feedback and proactive outreach [77] - Daily check-in reminders [77] - Home-based delivery [77] - Positive usability (SUS) [80] - Live supervised group sessions [81] - High enjoyment and usability [81] - Flexible, self-directed structure [78]   Qualitative*^c^*   - Flexible Goal reminders [30] - Flexibility to incorporate NUDGE into daily schedule [20] - Clear instructions [26] - Gradually increasing task difficulty [26] - Encouragement [26] - Design and features of the immersive app [26] - Tracking and monitoring [26,28] - Fun and engaging activities [27] - Receiving a free Fitbit [24] - Activity tracker [21] - Peer comparison [28] - Competition component [28] - Goal setting [28,29] - Activity logging [29] - Information on local activity resources[29] - Incentives [31] - Variety of videos and sports [20,22] - Game-based scoring and competition [84] - Accessibility [80] - Weekly phone calls and app reminders [80] - Goal-setting feature (choice of goals) [78]   **Barriers**  Statistically^a^   - Duration of the study [20,22]   Descriptive*^b^*   - Social features [26] - Technical difficulties [23,27] - Synchronization issues[27] - Dissatisfaction with equipment [23,27] - Duration of the study [28] - Weekly challenges and Facebook videos [28] - Receipt of text messages during class time as well as frequency and volume of notifications [28] - Excessive video duration [21] - Educational modules [25] - Device technical issues (charging, syncing) [83] - Lower retention than usual care [83] - Unattractive device; synchronization difficulties [84] - Limited customization options [79] - Low use of group-challenge feature [82] - Competitive leaderboards discouraging for lower-ranked [82] - Virtual peer interaction lacks in-person support [82] - VR headset technical problems and side effects [81] - Absence of professional/therapist guidance [78]   Qualitative*^c^*   - Dissatisfaction with equipment [27,28,30], - Not being able to choose between different goals and activities[26] - Inaccuracy of the device [27,28,30] - Technical difficulties [24,28,29,31,32], - Available competing systems and informations [24,28] - Not appealing website content [24,29] - Synchronization issues [24,28,29,31,32] - Message board [29] - Expert forum [29] - Website: too much writing and not “youthful” enough [29] - Program: not social enough [29] - Logistical barriers (charging, logging into website) [24,28,29,31,32] - No ease of use [29,31] - Not rewarding and engaging enough [31] - Wish for reminders [32] - E-mail instead of text messages [32] - Absence of social interaction in virtual mode [86] - Forgetting to reattach device after charging [84] - Skin irritation from device wear [84] - Device fails to register some activities [84] - Inflexible step goals (no adjustment for off days) [84] - Insufficient reminders [84] - Technical issues (app malfunction, app deleted) [80] - Overwhelming content volume; wanted personalization [78] | **Facilitators**  Statistically^a^   - Being Hispanic [20]   Descriptive*^b^*   - Reasons for playing: fun for boys, boredom for girls [33] - Step-count feedback not burdensome [83] - Perceived competence, autonomy and relatedness [85]   Qualitative*^c^*   - Personal benefits [26] - Family involvement [28] - Parent-child buddy system [84] - Perceived health benefits [80] - Positive emotional and family effects [78]   **Barriers**  Descriptive*^b^*   - Injury, illness and pain [23] - Forgetting to wear the tracker [83] - Fear of hypoglycemia [79] - Lack of disease-related information [79] - COVID-19 distancing and winter break [82] - Scheduling difficulties and waning motivation [77] - Unwillingness to continue [81] - Health-related interruptions (hospitalisation, illness) [81]   Qualitative*^c^*   - No interest [26,27] - Not enough time [23,26] - Short-term motivation [28] - Limited access to Wi-Fi or mobile data plans [28] - Forgetting to use it [31,32] - Limited storage [32] - Fatigue, pain and reduced energy [80] - Difficulty maintaining regular activity [80] - Competing school demands [80]   Statistically^a^   - Low intention to reuse virtual exercise videos [86] - Poorer baseline sleep hygiene and lower baseline activity [78] |

Evidence types are indicated as follows: ^a^Relationships confirmed through inferential statistical testing (p < .05); ^b^Patterns identified through descriptive statistics without significance testing; ^c^Findings from participant feedback and researcher observations.

**Table S2. Factors influencing adherence in the health domain of obesity.**

| Intervention-related Factors | User-related Factors |
| --- | --- |
| **Facilitators**  Statistically^a^   - Communication with health coach [34,35] - Voting on social poll [35] - Parental participation [35,36] - Online food journal [37] - Healthy eating messages and booster [38] - First half vs. last half of the intervention [36,41] - Primary recruitment sites [39] - Providing the program in class (vs. homework) [40]   Descriptive*^b^*   - Communication with health coach [39,42] - My workouts, My steps and My goals functions [43] - First half vs. last half of the intervention [37,39,44]   Qualitative*^c^*   - Video describing how to read nutrition label with interactive activity and “Rethink your drink” exercise [45] - 5-minutes exercise activity and learning how to measure heart rate [45] - Screen time calculator and activity on how screen time can affect health [45] - Daily encouragement through peer-appealing conversational agent with real-time feedback [46] - Rewarding game system [46] - Competition with peers [46] - Low threshold for communication with health care professional [46] - Daily reminders and assigned activities [39] - Distraction ideas to use during times of cravings [39] - Help with problem food elimination [39] - Weekly phone call with coach [39]   **Barriers**  Statistically^a^   - Self-esteem / stress management message [38] - AppAlone arm(vs. AppCoach arm) [39]   Descriptive*^b^*   - “My motivation” function[36] - The chosen self-monitoring tools[36]   Qualitative*^c^*   - Too little individualization and support [47] - Too little variety in exercises provided [47] - Wish for more in-person resources and group-exercises classes [47] - Messages too frequent and repetitive [36] - Too long and boring training [39] - Too many number of sessions and weeks [39] - Technical difficulties [34,39] - Teacher feedback: Too long lessons with too big amount of reading [34] | **Facilitators**  Statistically^a^   - Successful reducing BMI in the first 12 weeks of the program leads to better adherence to the digital maintenance treatment [47] - Intrinsic motivation as well as autonomy support and relatedness [44] - Healthier parenting practices [44] - Younger age [40,44] - Higher household income [44] - Female gender [40]   Descriptive*^b^*   - Lower annual household income and public insurance [39]   **Barriers**  Statistically^a^   - Authoritative parenting style (using tracking tools) [41,44] - Depression [41,44]   Descriptive*^b^*   - Greater weight and shape concerns [37] - Being white [37] - Report depressed mood [37] - Higher weight status [48] - Other interfering activities [48] - Too busy [48] - Too tired [48] - Being away from home [48]   Qualitative*^c^*   - Lost motivation [48] |

Evidence types are indicated as follows: ^a^Relationships confirmed through inferential statistical testing (p < .05); ^b^Patterns identified through descriptive statistics without significance testing; ^c^Findings from participant feedback and researcher observations

**Table S3. Factors influencing adherence in the health domain of diet.**

| Intervention-related Factors | User-related Factors |
| --- | --- |
| **Facilitators**  Statistically^a^   - Frequency of text messages (2-3x/week) [49]   Descriptive*^b^*   - Online resources and interactive quizzes [49] - High acceptability/usability and trust in the app's healthy-eating content [87] - Gamification (badges, personal/team goals, statistics, daily/weekly reports, social sharing) [87] - Slide-bar to indicate portion of a shared family meal (addressed cultural barrier) [87] - Pre-trial 2-day troubleshooting period resolved app problems in advance [87] - Weekly in-person staff sessions supported adherence (authors flag as limiting real-world generalisability) [87] - Push notifications made information easy to access [88] - Age-relatable, adolescent-specific messaging [88] - Perceived credibility of dietitian-approved content [88] - Concise, non-overwhelming message format [88] - Canteen-staff familiarisation/training + remote IT support eased delivery [89]   Qualitative*^c^*  Receiving music [51,52]   - Goal setting [51,52] - Tips and recipes [51,52] - Food diary [51,52] - Reminders [51,52] - Videos, language and layout [53] - Visual design and layout [52] - Feedback component [52] - Comfort with the device [52] - Website informative, attractive and easy to use [89] - Reward scheme created a motivating sense of competition [89]   **Barriers**  Descriptive*^b^*   - Duration of the study [50] - Too many notifications per day [88] - Adult-oriented messages reduced relevance for some [88] - Unmet feature requests (more recipes, social-media sharing) [88] - Two-step website registration a major barrier (step 1 79.3%, step 2 only 55.4%; urban 44.6% vs rural 83.3%) [89] - Companion app Android-only, limiting uptake (35/179, 19.6% downloaded) [89] - Technical problems linking canteen till data to website (security firewalls, mismatched pupil records) delayed implementation [89]   Qualitative*^c^*   - Too few applicable scenarios for students eating in the dining halls [53] - Too few videos [53] - Not able to pause or stop the videos any time [54] - Participants would have appreciated additional online programs for other issues (e.g. stress) [54] - Too long sessions [54] - Getting more rewards (songs) [52] - Feedback: Wish to enter all foods, not just fruit and vegetables [52] - Delay between claiming and receiving rewards; embarrassment of public (assembly) reward distribution [89] | **Facilitators**  Statistically^a^   - Higher education [50] - Higher healthy snack ratio and higher score for self-efficacy to eat healthy [50] - Lowest sensitivity to reward score [50] - Feeling competent to use the app [50] - Experiencing positive affect due to the app [50]   Qualitative*^c^*   - Pupils receptive to the reward-for-healthy-eating concept [89]   Descriptive*^b^*   - Teacher and family support enabled meal photos at home and school [87]   **Barriers**  Qualitative*^c^*   - Too busy with school work [52] - Lack of time [52] - Log-in difficulties: unfamiliar school email, activation emails to junk mail, forgotten passwords [89] - Forgetting about the scheme; limited school-day time to check points [89] - High-value rewards perceived unattainable within the timeframe [89]   Descriptive*^b^*   - Two ranked-response food-knowledge questions poorly understood (lowered completion) [88] |

Evidence types are indicated as follows: ^a^Relationships confirmed through inferential statistical testing (p < .05); ^b^Patterns identified through descriptive statistics without significance testing; ^c^Findings from participant feedback and researcher observations.

**Table S4. Factors influencing adherence in the health domain of tobacco use.**

| Intervention-related Factors | User-related Factors |
| --- | --- |
| **Facilitators**  Descriptive*^b^*   - Easy to use and interesting [55] - Working with the narrator [55] - Paid retention check-ins and surveys built in to limit differential attrition [94] - High sustained engagement with counselor-facilitated group chats [92] - Opt-in prompt embedded in the in-visit questionnaire raised interest vs automatic texts [91] - Multiple email reminder prompts (vs one) raised engagement in under-engaged subgroups [90] - Full EHR/clinical-workflow integration credited with high overall engagement (80.9%) [90] - Peer-written "pushed" text messages had the highest engagement of all components [93] - Peer-messaging engagement attributed to homophily (similarity of teen writers and readers) [93] - High overall retention presented as evidence the delivery model was feasible/acceptable [93]   Statistically^a^   - Greater perceived support than control (advice, helpful info, not alone, connected) [92]   **Barriers**  Statistically^a^   - Significant participant dropout on the designated 'quit day' [56] - Lower 1-month follow-up rate in intervention arm than controls [94]   Qualitative*^c^*   - Technological implementation problems [57]   Descriptive*^b^*   - General support (feeling cared for/encouraged) no better than control [92] - Attrition driven by platform patterns and low incentives; Instagram rule changes and handle changes broke contact [92] - Carriers auto-blocked ~half of automated outreach texts as spam [91] - Manual data aggregation and manual outreach created lag between visit and first contact [91] - Cessation-focused framing may not resonate with AYA who do not identify as addicted [91] - NRT access barriers: under-18 insurance denials, required physician call, parental disclosure on delivery [91] - Large drop-off from expressed interest to treatment connection [91] - Peer videos had the lowest engagement; embedded links and low production quality vs TikTok/Instagram [93] - Gamification engagement moderate; only a single storyline offered despite formative demand for several [93] | **Facilitators**  Descriptive*^b^*   - Higher grades and better school performance [57] - Less absenteeism [57] - Speaking mostly English at home [57] - Having access to internet at home [57] - Lower rebelliousness score [57] - Non-smokers [57] - Longer pre-quit time [56] - Two or more quit attempts [56]   Statistically^a^   - Black/African American youth more likely to engage after multiple prompts [90] - Non-susceptible never-smokers more likely to engage after multiple prompts [90]   **Barriers**  Descriptive*^b^*   - Smokers or ex-smokers [57] - Subscribers from the South (USA) [56] - Privacy concerns about texting the pediatric office (at school or near a parent) [91] - Poor treatment fit for infrequent users (over half used nicotine only 1-3 days/month) [91]   Statistically^a^   - Male gender predicts nonresponse (dropout) [94] - Black or multiracial race [94] - Greater baseline vaping frequency [94] - Lower confidence to quit vaping [94] - Higher nicotine dependence [94] - Higher perceived addiction to vaping [94] - Greater past-year internalizing problems [94] - Lower baseline cigar/cigarillo use [94] - Female gender associated with greater attrition [92] - Older age associated with greater attrition [92] - Using no/only one other tobacco product predicts less engagement after multiple prompts [90] |

Evidence types are indicated as follows: ^a^Relationships confirmed through inferential statistical testing (p < .05); ^b^Patterns identified through descriptive statistics without significance testing; ^c^Findings from participant feedback and researcher observations.

**Table S5. Factors influencing adherence in the health domain of alcohol use.**

| Intervention-Related Factors | User-related Factors |
| --- | --- |
| **Facilitators**  Descriptive*^b^*   - Frequency of text messages (1-3/weeks) [58] - Personalized / therapist-tailored feedback as an engagement component [96]   Statistically^a^   - In-app peer-to-peer "nudge" feature (associated with family completion) [97]   Qualitative*^c^*   - Attractive, up-to-date layout and gamification (avatars, rewards, stories, videos); familiar, accessible language [95]   **Barriers**  Descriptive*^b^*   - Booster sessions at home [59] - Baseline questionnaire / program length perceived as too long (lowest-scoring item, esp. younger adolescents) [95] - Text readability and minor technical issues needing improvement [95] - Over-long film-based lectures (8-15 min) failing to hold attention [96] - Required mid-program log-out break discouraged re-engagement [96]   Qualitative*^c^*   - Ineffective e-mails (spam folder) [59] - Logistical difficulties [59] - Repetitive / duplicated questionnaire content [95] - Technical / navigation glitches with video pop-up windows (accidental exit, could not return) [95] - Dyadic gating: one member (usually the teen) not completing a component blocks the partner's progress [97] | **Facilitators**  Statistically^a^   - Female gender [60] - Younger age [60] - Higher education level [60] - Being Spanish [59] - Binge-drinking status (higher-risk drinkers rated design, videos and difficulty more favourably and needed less assistance) [95] - Older adolescent age (more likely to recommend; rated program length as more appropriate) [95] - Parents' higher alcohol-communication self-efficacy (increased completion) [97] - Parents' positive expectations from alcohol communication (increased initiation) [97] - Higher household income (>$60,000 vs <=$60,000) (increased initiation) [97] - Parents' own recent alcohol use (increased initiation) [97] - Teens' perceived open communication with parents (greater retention; trend for completion) [97] - Younger teen age (16 vs 17) (greater retention and completion) [97] - Teen non-White racial/ethnic identity (greater retention) [97]   Descriptive*^b^*   - Female gender [58] - Younger age [58] - Higher maximum number of standard drinks per occasion [58] - Lower weekly alcohol consumption [59,60] - Being Dutch [60]   Qualitative*^c^*   - Binge drinkers engaged more with action-planning content [95]   **Barriers**  Descriptive*^b^*   - Beginning placements in companies [59] - School-related logistical problems [59] - Lower educational level [59]   Qualitative*^c^*   - Lack of time / being too busy; lost passwords [97]   Statistically^a^   - Parents' higher alcohol-communication self-efficacy (reduced initiation) [97] - Parents' own recent alcohol use (reduced retention) [97] - Teen lifetime sexual activity / intentions (lower retention and completion) [97] - Severe depressive symptoms (proposed as a reason for low adherence) [96] |

Evidence types are indicated as follows: ^a^Relationships confirmed through inferential statistical testing (p < .05); ^b^Patterns identified through descriptive statistics without significance testing; ^c^Findings from participant feedback and researcher observations.

**Table S6. Factors influencing adherence in the health domain of multicomponent lifestyle interventions.**

| Intervention-related Factors | User-related Factors |
| --- | --- |

| **Facilitators**  Statistically^a^   - High parent engagement [61] - Use of self-regulation and narrative component [62] - Periodic reminders via SMS and e-mail (effect of email higher than SMS) [63] - Higher app engagement correlated with better outcomes (dose-response) [98] - Team competition with caregiver, when perceived motivating, linked to higher logging (r=0.45, P=0.04) [104] - Financial incentive raised leaderboard-competition motivation (CommitFit$ 75.5 vs CommitFit 48.7, P=0.04) [104] - Video-based delivery (animation + athlete/role-model storytelling) vs static online pamphlets [107]   Descriptive*^b^*   - Comfort of the equipment [64] - Easy handling [65] - Incentives [66] - Learning new info [66] - Feedback on progress [66] - Earning points [67] - E-mails, quizzes, Puzzled Pattys and getting recipes [68] - SMS text message prompts, prizes, web-based feedback and quiz questions [69,70] - Length of the program (4 months) [69,70] - Social skills topic [69,70] - Quizzes and videos [68,69] - Interaction typically on weekdays from 3 PM to 1 AM or on weekends [71] - Gamified design ("health coins", unlockable "health village", medication "streaks") [98] - High perceived fun/acceptability of the app [98] - Weekly automated SMS summaries to parents [98] - Platform migration to a more stable system eliminated crashes/photo deletion [100] - Automated reminder emails for outstanding modules [107] - Shorter 1-week duration plus completion-contingent incentive (vs 5-week uncompensated pilot) [107] - Academic incentive (10% final-grade increase) driving extrinsic motivation and retention [108] - Low/moderate exercise intensity favouring retention [108] - Novel app-mediated format of promoting physical activity [108] - High usability and ease of use of self-monitoring web application [102] - Manualised, standardised content supporting consistent delivery [102]   Qualitative*^c^*   - Short baseline questionnaire [73] - Platform and structure [73] - Friendly health competitions [74] - Small altruistic rewards [74] - Motivational support [75] - Smartwatch reward mechanisms (step-goal fireworks, virtual medals) [99] - Detailed data feedback (sleep stages, calories) increased self-checking [99] - Forced sequential page completion + automated reminders (eliminated missing data) [99] - Informative, evidence-based content and engaging cartoon storylines/characters [99,101,103,110]   **Barriers**  Descriptive*^b^*   - Uncomfortable heart rate monitor [64] - Too few new information on website [66] - Duration of the study [61,68,71] - Contest prompt at Week 8 and smoking cessation stage-of-change assessment at Week 10 [72] - Picture and message contests [69,70] - Perceived burden of daily digital self-monitoring (app no better than paper) [101] - Device dependency (phone damage caused dropout) [98] - Digital divide / reliance on a supportive clinical framework [98] - Short intervention with no booster/continued contact (effects faded by 18 months) [105] - Smartwatch setup and use difficult and time-consuming [99] - Excludes adolescents without smartphones; school conflicts (exams, sports) [99] - Technical difficulties (missed notifications 67%, missed dietitian tips 73%, crashes 33%, photo deletion 20%) [100] - Notifications still blocked by the phone's own settings after the fix [100] - Absence of gamification features in the app [106] - Fixed, non-adaptive step target with no coaching support or nutrition component [106] - Sharp decline in app use over time (most stopped systematic use after week 6) [106] - Very low uptake of optional companion app (11.3%) and booster content (0.1%) [[99,101,103,110]] - Low app use limiting translation of knowledge into behaviour change [[99,101,103,110]]   Qualitative*^c^*   - Wrong timing of surveys [64] - Time consuming questionnaire at baseline[75] - Technical difficulties [75] - Modules too long; content repetitive/slow-paced; storylines unrealistic or lacking long-term outcomes [[99,101,103,110]] - Website glitches/slow loading, text overflowing borders, and longer-than-advised completion time (teacher-reported) [99,101,103,110]   Statistically^a^   - Gamified app (Pokémon Go) rated lower than pedometer app on information, usability and perceived impact (difficult interface, internet-dependent, inaccurate location) [108] | **Facilitators**  Statistically^a^   - Female gender [73] - Higher BMI [75] - Being British[73,75][73] - Weaker intentions to consume fruits and vegetables [73] - Less exercise at baseline [75] - Lower BMI [75] - In better health [75] - Mother in the dyad [61] - High parent education [61] - Younger age [76] - Having been drunk in the past month [63] - Having used the Internet for health reasons in the past [63] - Perceiving more benefits of quitting [72] - Caregiver app engagement correlated with adolescent engagement (r=0.65, P=0.002) [104]   Descriptive*^b^*   - Male gender [74] - PA exercises: from noon to 6 PM and mental health exercises from 6 AM to noon [74] - Higher risk perception of drugs [63] - Lower alcohol and tobacco consumption but having been drunk in the past month [63] - Less alcohol and drug abuse problems in family [63] - Female gender [69,70] - Younger age [69,70] - Non-problematic drinking [69,70] - Female gender and at upper secondary school [69,70] - Younger age (numerically higher weekly logins) [98] - Mother more acceptable co-participant than friend (49.0% vs 20.0% joined) [105] - Caregiver inclusion broadly acceptable (71.9/100, SD 22.3), regardless of incentive (P=0.46) [104]   Qualitative*^c^*   - Being part of something bigger (research project) [64] - Enjoy learning about health and challenge themselves[64] - Peer/"bandwagon" effect (invited classmates to exercise together) [99] - Self-comparison/self-evaluation using watch data [99] - Higher engagement tracked with self-perceived dietary change (80% [4/5] high- vs 33% [2/6] moderate-engagement) [100]   **Barriers**  Statistically^a^   - Adolescents of nonacademic vs. academic track [62] - Students with religious affiliation [63] - Older age [72] - Binge drinking [72] - Immigration background [72] - Lower paternal education in non-completers (P=0.003) [101] - Higher forced training volume degrading app perception across all uMARS dimensions (overweight/obese; mandatory grade-linked use) [108] - Higher training volume associated with problematic mobile phone use (normal-weight subgroup) [108]   Descriptive*^b^*   - Being too busy [66] - Using the app did not change behavior [62] - Subjectively, having already a healthy life [62] - Already using other apps and channels to track and improve lifestyle [62,63] - No motivation from environment [62] - No interest [63,68] - Lost password [68] - Extracurricular activities [63] - Missing at school [63] - Being in a relationship [63] - Prefer other channels [63] - Immigration background [69,70] - Lack of interest (most common dropout reason) [101] - Distance from home to clinic; mother starting work (could not accompany adolescent) [101] - Starting a weight-affecting medication [101] - Trend toward lower maternal education and family income in non-completers [101] - Lost interest [98] - Non-uptake: 37% never joined the offered program (1,922/5,260) [105] - Caregiver team-competition only weakly motivating overall (neutral, 51.8/100) [104] - Extrinsic/financial motivation not sustained after incentive withdrawn (90-120 days) [104] - Not attending postintervention measurements; voluntary withdrawal from the research [106] - Already-high baseline knowledge reducing perceived novelty of content (mental-health topic) [107] - Low comfort with Facebook group and low perceived usefulness of chat feature [102] - Low uptake of optional synchronous chat sessions (23.7% requested any) [102] - No baseline characteristic predicted retention [109]   Qualitative*^c^*   - Being too busy [74] - Not enough storage for the app [62] - Feels obligatory [62] - Reluctance to invite a friend (project felt "too private") [105] |
| --- | --- |

Evidence types are indicated as follows: ^a^Relationships confirmed through inferential statistical testing (p < .05); ^b^Patterns identified through descriptive statistics without significance testing; ^c^Findings from participant feedback and researcher observations.

**Appendix S3.**

**Table S7.** Physical Activity: Study Characteristics.

| Authors | Country | Setting | Delivery type | Level of personal support^1^ | Incentives^2^ | Number of participants | Mean age (SD)^3^ | | Intervention duration in days |
| --- | --- | --- | --- | --- | --- | --- | --- | --- | --- |
| Cushing et al., 2021 [30] | USA | Community-based | SMS + accelometer | N/A | 15$ + 25$ | 20 | 15.25 (1.8) | | 20 |
| Direito et al., 2015 [26] | New Zealand | Online | App (immersive) | N/A | None | 17 | 15.78 (1.11) | | 56 |
|  | New Zealand | Online | App (nonimmersive) | N/A | None | 16 | 15.69 (1.04) | | 56 |
| Dunton et al., 2016 [20] | USA | Online | App + accelometer | Continuous | 180$ | 39 | | 15.9 (1.2) | 14 |
| Ha et al., 2022 [27] | Australia | Online | App + activity tracker | Support messaging system for technical help and questions | None | 30 | | 10.2 (1.5) | 84 |
| Heale et al., 2018 [23] | Canada | Online | Misfit Flash™ tracker + smartphone | N/A | None | 31 | | 15.1 (N/A) | 28 |
| Howell et al., 2018 [119] | USA | Online | Internet | N/A | N/A | 53 | | 12.8 (N/A) | 168 |
| Jago et al., 2006 [120] | USA | Community-based | Internet | Continuous | None | 240 | | 13 (0.1) | 63 |
| Kaos et al., 2018 [22] | Canada | Home-based | Internet | N/A | None | 40 | | 10.83 (1.05) | 42 |
| Kaos et al., 2018 (2nd intervention arm) [22] | Canada | Home-based | Internet | N/A | None | 32 | | 10.16 (0.88) | 42 |
| Klausen et al., 2016 [121] | Denmark | Online | App + SMS + Internet | Continuous | None | 81 | | 13.7 (0.4) | 365 |
| Knox et al., 2019 [24] | UK | Online | Internet + activity tracker | N/A | None | 24 | | 10.4 (1.1) | 56 |
| Koorts et al., 2020 [28] | Australia | Online | Facebook + App + activity tracker | N/A | Free activity tracker | 144 | | 13.7 (0.4) | 84 |
| Larsen et al., 2018 [29] | USA | Online | Internet + accelometer | Continuous | None | 21 | | 14.7 (2.1) | 84 |
| Lau et al., 2012 [122] | Hong-Kong | Online | Internet + SMS | N/A | N/A | 38 | | 12.29 (0.87) | 56 |
| Lelieveld et al., 2010 [123] | Netherlands | Home-based | Internet | Continuous | None | 17 | | 10.6 (1.5) | 119 |
| Martínez-López et al., 2022 [33] | Spain | Online | App | None | None | 78 | | 13.56 (1.08) | 56 |
| Mateo-Orcajada et al., 2024 [106] | Spain | Online | Step tracker app | N/A | None | 240 | | 13.96 (1.21) | 70 |
| Meyer et al., 2021 [124] | Germany | Online | Internet | Continuous | None | 35 | | 13.08 (2.8) | 168 |
| Pope et al., 2018 [31] | USA | Online | Chromebook + Fitbit | Continuous | N/A | N/A | | N/A | 84 |
| Slootmaker et al., 2010 [125] | Netherlands | Online | Accelometer + Internet | N/A | None | 41 | | 15.35 (1.1) | 90 |
| Stephens et al., 2022 [25] | Canada | Online | App + Fitbit | Continuous | None | 15 | | 16.6 (1.2) | 84 |
| Tripicchio et al., 2023 [21] | USA | Home-based | Videos + SMS | Continuous | Prizes | 62 | | 12.4 (1.2) | 42 |
| Zapata et al., 2018 [32] | USA | Home-based | App | Continuous | None | 14 | | 15.3 (2.0) | 180 |
| Ahmadi et al., 2025 [86] | Canada | Home-based (asynchronous online videos) | Asynchronous exercise videos (pre-recorded; online) | None | N/A | 40 | | 15.5 (0.8) | N/A |
| Bicki et al., 2024 [83] | USA | Clinical | Activity tracker (Fitbit Flex 2) + personalized feedback (email/phone call) | Continuous | None | 42 | | 18 (N/A) | 182 |
| Friedman et al., 2024 [84] | USA | Home-based | Wearable accelerometer (Fitbit Inspire HR) + gamification platform (Way to Health), parent-child dyad | None (automated platform feedback/notifications only, no human coach) | Fitbit device retained (not returned) if full 24-week study completed; no monetary incentive reported | 27 | | 14 (N/A) | 84 |
| Gómez-Cuesta et al., 2024 [85] | Spain | School-based (app used after school hours; promoted via PE class) | App (Pokémon Go - gamified step tracker) | None (brief initial app orientation only; no ongoing support) | Non-monetary (up to 1 extra point on final PE grade) | 72 | | 13.76 (1.41) | 70 |
| Gómez-Cuesta et al., 2024 (2nd intervention arm) [85] | Spain | School-based (app used after school hours; promoted via PE class) | App (MapMyWalk - step tracker) | None (brief initial app orientation only; no ongoing support) | Non-monetary (up to 1 extra point on final PE grade) | 67 | | 13.76 (1.41) | 70 |
| Gómez-Cuesta et al., 2024 (3rd intervention arm) [85] | Spain | School-based (app used after school hours; promoted via PE class) | App (Pacer - step tracker) | None (brief initial app orientation only; no ongoing support) | Non-monetary (up to 1 extra point on final PE grade) | 70 | | 13.76 (1.41) | 70 |
| Gómez-Cuesta et al., 2024 (4th intervention arm) [85] | Spain | School-based (app used after school hours; promoted via PE class) | App (Strava - step tracker) | None (brief initial app orientation only; no ongoing support) | Non-monetary (up to 1 extra point on final PE grade) | 71 | | 13.76 (1.41) | 70 |
| Hormazábal-Aguayo et al., 2025 [79] | Spain | Home-based (app used independently; recruited via a hospital Pediatric Endocrinology Unit) | App (personalized muscle-strengthening exercise sessions + glucose monitoring/guidance + gamification) | None | Gamification only (in-app experience points and ranking system); no monetary incentives | 29 | | 13.15 (2.43) | 168 |
| Kwon et al., 2025 [82] | USA | School-based | Wearable activity tracker (Fitbit Inspire 2) + app + family group challenge | Continuous (research staff feedback/encouragement + parent involvement via shared dashboard) | Non-monetary (Fitbit Inspire 2 device given as compensation, no USD value stated) | 22 | | 13.3 (0.5) | 42 |
| Kwon et al., 2025 (2nd intervention arm) [82] | USA | School-based | Wearable activity tracker (Fitbit Inspire 2) + app + peer group challenge | Continuous (research staff feedback/encouragement + peer/classmate leaderboard interaction) | Non-monetary (Fitbit Inspire 2 device given as compensation, no USD value stated) | 26 | | 13.2 (0.4) | 42 |
| Li et al., 2026 [77] | China | Home-based | App (WeChat platform) + nurse-led remote coaching | Continuous | 1200 CNY (~$180 USD) completion stipend | 34 | | 11.2 (2.7) | 84 |
| Miskin et al., 2026 [80] | India | Home-based | App (PhysiApp) + weekly phone calls | Weekly phone calls + app-based reminders (periodic, not continuous) | N/A | 30 | | 11.6 (4.1) | 28 |
| Ozyemisci Taskiran et al., 2025 [81] | Turkey | Home-based (telehealth via videoconference) | VR headset (Meta Quest 2) exergames (Beat Saber; Les Mills Bodycombat; All-In-One Sports; Ohshape) + live videoconference-supervised group tele-exercise | Continuous | None | 17 | | 14 (N/A) | 84 |
| Ozyemisci Taskiran et al., 2025 (2nd intervention arm) [81] | Turkey | Home-based (telehealth via videoconference) | Live videoconference-supervised group tele-exercise (video-guided calisthenics/aerobic/strength exercises, no VR) | Continuous | None | 17 | | 16 (N/A) | 84 |
| Smith et al., 2026 [78] | Canada | Online (self-directed; home/community use; recruited via outpatient/community mental health services) | Web-based multi-module e-resource (Know Your Habits, Physical Activity, Screen Time, Sleep sections; self-selected Learn / Pros-and-Cons / SMART Goals content) + optional access to a registered kinesiologist | Minimal/optional (self-directed; optional kinesiologist on request; weekly telephone check-ins were feasibility monitoring, not treatment support) | N/A | 23 | | 15.7 (1.7) | 90 |

^1^Personal support categorized as: none, continuous, or not applicable (N/A); detailed descriptions provided for cases outside these categories. ^2^Monetary incentives converted to USD. ^3^Age reported as available when mean and standard deviation not provided in original article.

**Table S8.** Physical Activity: Adherence-related Study Characteristics.

| Authors | NSessions | | Intended use | | Reported adherence | | Rate of intervention completion^1^ | | | Adherence rate^2^ | Attrition |
| --- | --- | --- | --- | --- | --- | --- | --- | --- | --- | --- | --- |
| Cushing et al., 2021 [30] | N/A | | Daily interactions with SMS | | average 1.35 participant responses per day; average 1.66 messages sent by NUDGE per day; average 3 total exchanged messages per day; small, but significant decline in participant engagement with the NUDGE bot across time | | N/A | | | 100% | 0% |
| Direito et al., 2015 (2nd intervention arm)     [26] | N/A | | Use app 3 times a week and work through each workout | | two-thirds of participants in the intervention groups reported using the app either 2 (31%) or 3 times per week (31%), whereas 8 of 32 (25%) only used it 1 time per week | | 31% | | | N/A | 0% |
|  | N/A | | Use app 3 times a week and work through each workout | | two-thirds of participants in the intervention groups reported using the app either 2 (31%) or 3 times per week (31%), whereas 8 of 32 (25%) only used it 1 time per week | | 31% | | | N/A | 6.25% |
| Dunton et al., 2016 [20] | N/A | | N/A | | 80.5% average compliance rate to EMA prompts; 98.5% survey completion rate once started; 82.3% of completed surveys responded to after first prompt | | N/A | | | 80.5% | 9.1% |
| Ha et al., 2022 [27] | 10 | | 10 modules | | 90% completed baseline assessments; 77% (23) commenced iBounce program; 83% (19/23) completed the intervention; 57% (13/23) completed all 10 modules; 79% (15/19) activity tracker compliance | | 57% | | N/A | | 30% |
| Heale et al., 2018 [23] | N/A | | Wearing accelometer 24h per day, 7 days a week for 28 consecutive days | | On average, participants had activity logged on their Misfit Flash™ application for 72% of the days in the intervention period. 19% wore their Misfit Flash™ every day of the study period. 58% wore the Misfit Flash™ for at least 80% of the days of the study period. | | 19% | | 72% | | 6.45% |
| Howell et al., 2018 [119] | N/A | | N/A | | The completion rate for the intervention group was slightly higher than the completion rate in the control group (84.2% vs 80.6%, respectively) | | 84.2% | | N/A | | 15.9% |
| Jago et al., 2006 [120] | N/A | | Log in at least twice a week | | 75% on intervention participants logged onto the website at least once per week | | N/A | | N/A | | 9.6% |
| Kaos et al., 2018 [22] | 30 | | Play the game as frequently as they could | | Mean play session duration was 37.65 (SD = 15.39) min/day. Mean play session duration was M  =  40.97 (SD  =  15.50) min/day in Week 1 and M = 35.57 (SD = 13.55) min/day in Week 6; the decrease across time was significant; mean play duration for participants was 133.45 (SD = 81.27) min in Week 1. In Week 6, mean play duration was still 77.23 (SD = 84.09) min | | N/A | | N/A | | N/A |
| Kaos et al., 2018 (2nd intervention arm) [22] | 30 | | Play the game as frequently as they could | | Mean play session duration was 37.65 (SD = 15.39) min/day. Mean play session duration was M  =  40.97 (SD  =  15.50) min/day in Week 1 and M = 35.57 (SD = 13.55) min/day in Week 6; the decrease across time was significant; mean play duration for participants was 133.45 (SD = 81.27) min in Week 1. In Week 6, mean play duration was still 77.23 (SD = 84.09) min | | N/A | | N/A | | N/A |
| Klausen et al., 2016 [121] | N/A | | Registration of PA via the application for at least 2 consecutive weeks; 60 min daily activity | | Only 8 (10%) patients were active users during the last week of the intervention. 57 (70%) of the patients in the IG adhered to the intervention using the application for at least two consecutive weeks; 30% did not adhere | | 70% | | N/A | | 28.4% |
| Knox et al., 2019 [24] | N/A | | Website visits and synchronizing accelometer times a week | | Participants synchronized their watches on 48 out of the required 144 occasions (33.3%; weekly, weeks one-seven of intervention) | | N/A | | 33.3% | | 24.5% |
| Koorts et al., 2020 [28] | N/A | | Completion of weekly challenges and Facebook engagement | | 18.6% wore Fitbit daily in last week of program; 35.5% reported not wearing it at all in last week; 36.1% reported completing weekly challenges; 22.4% (13/58) of females and 15.0% (9/60) of males wore the Fitbit on all 7 days | | 36.1% | | N/A | | 7% |
| Larsen et al., 2018 [29] | N/A | | N/A | | After 1 month, 81% (17/21) completed a check-in call; Moderate adherence; 63% of monthly questionnaires completed; 42% reported reading most/all tailored reports; 42% reported reading at least half of emailed tip sheets | | N/A | | N/A | | 10% |
| Lau et al., 2012 [122] | 4 (N/A) | | Access Internet program twice per week and read daily SMS on weekdays | | Mean weekly login rate 0.5 times/person; 79% read an average of 1.3 SMS/person/week; 47% replied to ~3.8 SMS/person | | N/A | | N/A | | 0% |
| Lelieveld et al., 2010 [123] | 17 | | Completion of weekly online sessions, attendance at group sessions | | 82% completed full internet program, 18% completed most but not all | | 82% | | N/A | | 0% |
| Martínez-López et al., 2022 [33] | N/A | | N/A | | Percentage of participants actively playing Pokémon GO (47.6%); Average daily playtime (40 min/day); | | 47.6% | | N/A | | 12.3% |
| Mateo-Orcajada et al., 2024 [106] | N/A | | EG participants used a step tracker app a minimum of 3 times per week, covering an incremental distance from 7000 steps per day (week 1) to 12,500 steps per day (week 10). | | 0-25% of training volume completed by 129 (53.75%) of participants; 25-50% of training volume completed by 39 (16.25%) of participants; 50-75% of training volume completed by 33 (13.75%) of participants; 75-100% of training volume completed by 39 (16.25%) of participants | | N/A | | N/A | | N/A |
| Meyer et al., 2021 [124] | 13 | | 72 sessions | | actual mean weekly exercise adherence was 33% | | N/A | | 33% | | 13% |
| Pope et al., 2018 [31] | N/A | | N/A | | 36% never wore Fitbit; 50% wore Fitbit <10 days; Only 21 students played at least one game | | N/A | | N/A | | 79% |
| Slootmaker et al., 2010 [125] | | N/A | | N/A | | 65% of the PAM users reported to have worn the PAM regularly or often. 56% of the PAM users uploaded their PAM to the PAM COACH with a median of 5 times (inter-quartile range: 2–8 times) in 3 months. 24% (n = 10) uploaded the PAM every 2 weeks. Only 23% of the PAM users set a personal goal (all higher than the standard goal) and 35% (n = 9) entered their favourite activities on the site. The tailored advice was read by 38% of the PAM users. | | N/A | | N/A | 21.8% |
| Stephens et al., 2022 [25] | | 12 | | Completion rate of ≥80% of all possible coaching sessions, educational modules and accelometer wear days combined | | 80% of coaching calls completed; 89% Fitbit wear adherence; 42% of educational modules completed; Average 61 in-app messages exchanged with coaches | | N/A | | 70.3% | 13% |
| Tripicchio et al., 2023 [21] | | N/A | | N/A | | 41.2% reported doing 3-4 workouts per week; 91.2% reported watching the workout videos during the program; The average percentage viewed was only 16.8% and the average view duration was only 4:16 min | | N/A | | 16.8% | N/A |
| Zapata et al., 2018 [32] | | 78 | | Completion of 3 sessions per week | | One participant had 100% HEP adherence. The remaining 12 participants either did not use the app or less than once per week. | | 7.6% | | N/A | 33.3% |
| Ahmadi et al., 2025 [86] | | N/A | | N/A | | 0/28 (0%) did not watch or participate; 0/28 (0%) watched without participating; 8/28 (28.6%) did part of a workout session; 20/28 (71.4%) did a whole workout session (recalculated using n=28; paper's listed 14.04%/35.09% appear to use combined n=57 as denominator, p. 112) | | 71.4% | | N/A | N/A |
| Bicki et al., 2024 [83] | | N/A | | Wear Fitbit daily; feedback triggered if steps below age-referenced goal (10,000/day adults/adolescents; 12,000/day school-age children) | | bimodal adherence: ~28% wore device 0 days/week, 50% wore device 7 days/week (averaged across assessments); baseline median step count 8900/day; 29% at step goal at baseline; 88% (22/25) rated device easy to use; 65% reported frequently forgetting to wear it; 96% (25/26) would recommend intervention; only 8% found feedback excessive | | 62% | | N/A | 38% |
| Friedman et al., 2024 [84] | | N/A | | Wear Fitbit daily and meet daily step goal as designated team representative at least 5 of 7 days/week to advance a game level | | Fitbit worn for 95% of participant-days during the 12-week intervention (95% CI 94-96) and 81% during the 12-week follow-up (95% CI 79-82); by dyad member: adolescent survivors 93% (95% CI 92-94) intervention / 79% (95% CI 77-81) follow-up, parents 97% (95% CI 96-97) intervention / 83% (95% CI 81-84) follow-up; prespecified step goal met on 64% (95% CI 63-66) of days during intervention (59% children, 70% parents) and 37% (95% CI 35-38) during follow-up (32% children, 42% parents); at end of 24-week study 17/23 dyads responded to the AIM acceptability survey, 82% of survivors (14/17) and 94% of parents (16/17) rated intervention as acceptable | | N/A | | 95% (Fitbit worn, 12-week intervention; 95% CI 94-96, p. 3-4) | N/A |
| Gómez-Cuesta et al., 2024 [85] | | N/A | | Use app >=3 times/week for 10 weeks; per-session distance target increasing from 4.57km (week 1) to 8.00km (week 10) | | Average total distance traveled over 10 weeks: 146.91+/-14.35 km; no significant difference in distance traveled between the 4 apps (p=0.657); 3/72 (4.17%) withdrew during the intervention and were not measured at post-test; % of participants meeting the weekly distance/frequency target not reported | | 95.83% | | N/A | 4.17% |
| Gómez-Cuesta et al., 2024 (2nd intervention arm) [85] | | N/A | | Use app >=3 times/week for 10 weeks; per-session distance target increasing from 4.57km (week 1) to 8.00km (week 10) | | Average total distance traveled over 10 weeks: 144.53+/-17.65 km; no significant difference in distance traveled between the 4 apps (p=0.657); 2/67 (2.99%) withdrew during the intervention and were not measured at post-test; % of participants meeting the weekly distance/frequency target not reported | | 97.01% | | N/A | 2.99% |
| Gómez-Cuesta et al., 2024 (3rd intervention arm) [85] | | N/A | | Use app >=3 times/week for 10 weeks; per-session distance target increasing from 4.57km (week 1) to 8.00km (week 10) | | Average total distance traveled over 10 weeks: 148.64+/-19.42 km; no significant difference in distance traveled between the 4 apps (p=0.657); 3/70 (4.29%) withdrew during the intervention and were not measured at post-test; % of participants meeting the weekly distance/frequency target not reported | | 95.71% | | N/A | 4.29% |
| Gómez-Cuesta et al., 2024 (4th intervention arm) [85] | | N/A | | Use app >=3 times/week for 10 weeks; per-session distance target increasing from 4.57km (week 1) to 8.00km (week 10) | | Average total distance traveled over 10 weeks: 148.27+/-16.83 km; no significant difference in distance traveled between the 4 apps (p=0.657); 2/71 (2.82%) withdrew during the intervention and were not measured at post-test; % of participants meeting the weekly distance/frequency target not reported | | 97.18% | | N/A | 2.82% |
| Hormazábal-Aguayo et al., 2025 [79] | | N/A | | ≥3 muscle-strengthening sessions/week (ADA/ISPAD-based target used as the study's adherence benchmark, p. 4 Table 1 footnote) | | mean sessions per week 3.13 (SD=1.40); only 3/27 (11.1%) met the ≥3 sessions/week recommendation; app usage self-reported | | 93.1% (27/29 completed the intervention; 2 did not complete, p. 3) | | N/A | 6.9% (2/29) during the intervention period |
| Kwon et al., 2025 [82] | | N/A | | Wear Fitbit and self-monitor PA throughout the 6-week intervention; weekly goal-setting on Mondays after motivational video; ongoing family Fitbit challenge to share daily steps/MVPA with a parent | | Fitbit device usage 61% often/almost always; mobile app usage (exercise induction) 22% often/almost always; mobile app checked for group challenge 22% often/almost always; satisfaction: Fitbit 78%, mobile app 78%, group challenge feature 33% | | 63.6% (14/22; derived from Fig. 1 CONSORT diagram, p. 4; paper states FCG retention 64%, p. 3) | | 61% (Fitbit device usage, often/almost always) | 36.4% (8/22; p. 4) |
| Kwon et al., 2025 (2nd intervention arm) [82] | | N/A | | Wear Fitbit and self-monitor PA throughout the 6-week intervention; weekly goal-setting on Mondays after motivational video; Thursday/Friday review of activity progress and leaderboard within peer group | | Fitbit device usage 56% often/almost always; mobile app usage (exercise induction) 44% often/almost always; mobile app checked for group challenge 56% often/almost always; satisfaction: Fitbit 83%, mobile app 89%, group challenge feature 50% | | 69.2% (18/26; derived from Fig. 1 CONSORT diagram, p. 4) | | 56% (Fitbit device usage, often/almost always) | 30.8% (8/26; p. 4) |
| Li et al., 2026 [77] | | 48 | | 4 sessions per week, 45-60 min per session | | 84.4% mean adherence to prescribed exercise sessions among HeartFIT participants who completed follow-up; 2/34 (5.9%) had poor adherence (<80% of sessions), excluded from per-protocol analysis; 32/34 (94.1%) completed follow-up (2 lost to follow-up: 1 lost contact, 1 withdrawal); 2 additional participants missed only the in-person CPET due to COVID-19 restrictions but completed remote questionnaires | | N/A | | 84.4% | 5.9% |
| Miskin et al., 2026 [80] | | 12 | | Exercise program: 20-30 min/session, 3 days/week (alternate days) over 4 weeks; adherence defined a priori as participation in a minimum of 8 of 12 exercise sessions | | Adherence to the m-Health exercise program was low, with 18.5% meeting the ≥8/12-session criterion; session-count distribution (N=27): 8-12 sessions 5 (18.5%), 6-7 sessions 6 (22.2%), 4-5 sessions 3 (11.1%), 1-3 sessions 13 (48.1%); "very few participants attend[ed] ≥8 exercise sessions"; usability rated Best imaginable/Excellent by 14/27 (51.9%) and satisfaction reported by 70.3% of parents and 62.9% of participants | | 90% | | 18.5% | 10% |
| Ozyemisci Taskiran et al., 2025 [81] | | 36 | | Attend supervised group tele-exercise sessions 3x/week, 30 min/session, for 12 weeks | | median attended sessions 28 of 36 (78%); 64% attended >=70% of sessions; median % of target heart rate achieved during sessions 106% (IQR 19%) | | 64.71% | | 78% | 35.29% |
| Ozyemisci Taskiran et al., 2025 (2nd intervention arm) [81] | | 36 | | Attend supervised group tele-exercise sessions 3x/week, 30 min/session, for 12 weeks | | median attended sessions 29 of 36 (81%); 60% attended >=70% of sessions; median % of target heart rate achieved during sessions 104% (IQR 16%) | | 58.82% | | 81% | 41.18% |
| Smith et al., 2026 [78] | | N/A | | N/A (self-directed; no developer-specified minimum interaction stated; a post-hoc analytic threshold of ≥10 webpage visits/3 months per behavior section was used only to categorize low-vs-high engagement, not as an intended-use target, p. 5) | | Among the 10 study completers: 70% (7/10) accessed the HELP e-resources on >10 days during the 3-month access period; 50% (5/10) achieved >10-day engagement with each of the screen time, sleep, and physical activity modules (not necessarily the same individuals across domains); 30% (3/10) engaged with all three behavior modules on >10 days, 20% (2/10) engaged with two domains, 20% (2/10) engaged with one domain, and 30% (3/10) did not exceed 10 days of access for any single lifestyle behavior; participation in weekly telephone check-ins was limited, precluding quantitative analysis of standardized feedback | | 43.5% | | N/A | 56.5% |

^1^Proportion of participants who completed the entire intervention. ^2^Overall intervention adherence rate or mean adherence rate across all intervention components.

**Table S9.** Diet: Study Characteristics.

| Authors | Country | Setting | Delivery type | Level of personal support^1^ | | Incentives^2^ | Number of participants | Mean age (SD) ^3^ | Intervention duration in days |
| --- | --- | --- | --- | --- | --- | --- | --- | --- | --- |
| De Cock et al., 2018 [50] | Belgium | Online | App | Continuous | | No | 522 | 14.96 (0.1) | 28 |
| Haas et al., 2017 [49] | USA | Online | SMS | None | None | | 31 | 15.8 (2.4) | 90 |
| Hutcheson, 2013 [51] | USA | Online | Software (MyPal) | N/A | | 10$ for each assessment | 18 | N/A | 28 |
| Monroe et al., 2015 [53] | USA | University-based | Internet | N/A | | Extra course credit | 241 | 18.81 (0.97) | 35 |
| Nicolaou et al., 2022 [54] | Republic of Cyprus | Online | Internet | N/A | | None | 62 | 15.27 (2.26) | N/A |
| Nollen et al., 2013 [52] | USA | Online | Software (MyPal) | N/A | | None | 15 | 11.9 (2.0) | 7 |
| Nollen et al., 2013 (2nd intervention arm) [52] | USA | Online | Software (MyPal) | N/A | | None | 13 | 10.8 (1.5) | 14 |
| Pedersen et al., 2016 [126] | Denmark | School-based | SMS | N/A | | Prize for class with highest participation | 489 | 12.9 (N/A) | 77 |
| Pedersen et al., 2016 (2nd intervention arm) [126] | Denmark | School-based | SMS + camp | One lecture from a dietician | | Prize for class with highest participation | 497 | 12.9 (N/A) | 77 |
| Braga et al., 2024 [87] | Vietnam | School-recruited; app used at home and school | App (gamified) - AI-based photo dietary assessment + game elements (goals, badges, scores, daily/weekly reports, team/social features) | Weekly in-person check-in/troubleshooting sessions | | None | 18 | Not reported (eligibility age range 12-18 years) | 28 |
| Braga et al., 2024 (2nd intervention arm) [87] | Vietnam | School-recruited; app used at home and school | App (non-gamified/limited) - AI-based photo dietary assessment only | Weekly in-person check-in/troubleshooting sessions | | None | 18 | Not reported (eligibility age range 12-18 years) | 28 |
| Gilliland et al., 2026 [88] | Canada | School-based | App (time-based + location-based push notifications) | None | | $15 CAD per survey completed, max $30 CAD (approx. $11-11.5 USD / approx. $22-23 USD) | 59 | 15.4 (0.8) | 56 |
| Moore et al., 2024 [89] | UK (Northern Ireland) | School-based | Website + reward-points scheme linked to cashless canteen till data (App available for Android only) | None | | Points-based rewards (stationery; sports equipment; vouchers; electronic items; top prize approx. GBP100 [approx. USD145] headphones) | 90 | N/A (age range 12-14 years; no mean/SD reported) | 120 |
| Moore et al., 2024 (2nd intervention arm) [89] | UK (Northern Ireland) | School-based | Website + reward-points scheme linked to cashless canteen till data (App available for Android only) | None | | Points-based rewards (stationery; sports equipment; vouchers; electronic items; top prize approx. GBP100 [approx. USD145] headphones) | 233 | N/A (age range 12-14 years; no mean/SD reported) | 120 |

^1^Proportion of participants who completed the entire intervention. ^2^Overall intervention adherence rate or mean adherence rate across all intervention components.

**Table S10.** Diet: Adherence-related Study Characteristics.

| Authors | NSessions | Intended use | Reported adherence | Rate of intervention completion^1^ | Adherence rate^2^ | Attrition |
| --- | --- | --- | --- | --- | --- | --- |
| De Cock et al., 2018 [50] | N/A | N/A | The mean exposure to the intervention, measured in the number of days that the adolescents logged into the app, was 4·78 (SD 6·21) for the full intervention group (n 416). When we excluded the adolescents who did not use the app (n 148), the mean exposure was 7·41 (SD 6·35) d; 99.2% logged in week 1, 56.7% week 2, 33.2% week 3, 20.5% week 4; The percentage of adolescents who logged in at each day of the intervention decreased gradually from day 1 until day 28. | N/A | 17% | 20.3% |
| Haas et al., 2017 [49] | 45 | 100% text message response | mean bidirectional text message response rate of 81% | N/A | 81% | 3.2% |
| Hutcheson, 2013 [51] | 6 | N/A | 81.1% daily compliance; 88.9% used the handheld computer; Of days that were captured, participants interacted with the program, defined as responding to at least one program-initiated prompt on 81.1% of those days; of the 6 total program prompts, 9.1% of days (N = 35) had perfect  compliance, 45.3% (N = 175) had 67% compliance or  better and 59.8% (N = 231) had 50% compliance or better. On average, daily compliance was 50.4% for all program prompts and 53.6% for main program prompts, which consisted of the two real-time reminders and the two real-time goal assessment and feedback prompts. | N/A | 81.1% | 0% |
| Monroe et al., 2015 [53] | 4 | 4 modules | 78.1% completed all modules; 10.2% accessed three modules; 7% accessed two modules; 4.7% accessed one module; | 78.1% | N/A | 22.4% |
| Nicolaou et al., 2022 [54] | 6 | 6 modules | 93% entered and completed pre-intervention measures and session 1, 66% completed session 2, 59% completed session 3, 53% completed session 4, 51% completed session 5 and 50% completed session 6 | 50% | N/A | 46.55% |
| Nollen et al., 2013 [52] | 28 | Completion of 28 prompts | They completed an average of 47.3% of the 28 prompts (4 prompts/day 7 days), with use ranging from 20.7% to 72.4% | N/A | 47.3% | N/A |
| Nollen et al., 2013 (2nd intervention arm) [52] | 84 | Completion of 84 prompts | Girls completed an average of 78.3% of the 84 prompts (6 prompts/day x 14 days), with use ranging from 43.3% to 97.6% | N/A | 78.3% | N/A |
| Pedersen et al., 2016 [126] | Daily SMS for 5 weeks | Interaction with SMS | The proportion of the possible participant SMSs sent varied widely between schools, from 30% to 70%. In the SMS group, individual schools sent from 30% to 70%; and, in the SMS/education group, from 33% to 66% of the possible SMSs; Engagement dropped over time (from 76% to 41% for boys from weeks 1-5 and 91.6% to 60.1% for girls) | N/A | 30-70% | N/A |
| Pedersen et al., 2016 (2nd intervention arm) [126] | Daily SMS for 5 weeks | Interaction with SMS | The proportion of the possible participant SMSs sent varied widely between schools, from 30% to 70%. In the SMS group, individual schools sent from 30% to 70%; and, in the SMS/education group, from 33% to 66% of the possible SMSs. | N/A | 33-66% | N/A |
| Braga et al., 2024 [87] | N/A | Record all meals/snacks consumed daily via photo (or manual entry) throughout the study; a priori adherence target of >=70% completed food records | Randomized pilot period (first 4 wk): 439/540 participant-days with completed food records, adherence 81%; whole-study exploratory figure (36 participants pooled, 6 wk): 1304/1548 participant-days, 84%; feasibility period (wk 5-6, both original groups now using gamified FRANI, not split by group): 412/504 participant-days, 82%; percentage of meals recorded (whole study) 97%; slight downward trend in adherence toward the end of the study period | 100% (inferred; no participant losses reported, CONSORT diagram states all 36 recruited were enrolled and retained, p. 2, 7, 10) | 81% | 0% (not explicitly reported) |
| Braga et al., 2024 (2nd intervention arm) [87] | N/A | Record all meals/snacks consumed daily via photo (or manual entry) throughout the study; a priori adherence target of >=70% completed food records | Randomized pilot period (first 4 wk): 473/540 participant-days with completed food records, adherence 88%; whole-study exploratory figure (36 participants pooled, 6 wk): 1304/1548 participant-days, 84%; feasibility period (wk 5-6, both original groups now using gamified FRANI, not split by group): 412/504 participant-days, 82%; percentage of meals recorded (whole study) 97% | 100% (inferred; no participant losses reported, CONSORT diagram states all 36 recruited were enrolled and retained, p. 2, 7, 10) | 88% | 0% (not explicitly reported) |
| Gilliland et al., 2026 [88] | N/A | Max 3 time-based messages/day + max 5 location-based (GPS-triggered) messages/day for 8 weeks | 53.7% interacted with app at least once/week; 38.9% clicked links in-app at least once/week; 74.1% said app benefitted them; 92.6% would recommend to a friend; 67.4% suggested improvable features; 31% liked the information most; survey-section completion rates: demographics 98.8%, food preferences 93.2%, nutrition perceptions 100%, food intake 99.8%/98.9%, participant experience 94.7%, food knowledge 85% (84.5%/85.6%), food purchasing 83.8%/76.5% (weekend subsection 67.5%/51.7%) | 91.5% | N/A | 8.5% |
| Moore et al., 2024 [89] | N/A | Register on the E4T website (2-step sign-up) and purchase higher-point (healthier) food/drink options in the school canteen on most days over the approx. 4-month scheme to accumulate points; top-tier reward required the healthiest breakfast/lunch choices on most days of the intervention, lowest-tier reward required healthier purchases consistently for approx. 2 weeks | Step 1 (website sign-up) completed by 87.8% (79/90) of consenting pupils; Step 2 (full registration/account activation) completed by 83.3% (75/90) (Table 4, p. 10); of all 85 rewards claimed across both intervention schools, 66 (78%) were claimed by pupils at this school; 29 pupils (71% of all reward-claimers across both schools) claimed at least one reward, i.e. 36.6% of this school's registered pupils; 14/43 (32.6%) males and 15/32 (46.9%) females claimed rewards (Table 5, p. 11-12) | N/A | 83.3% | 28.9% |
| Moore et al., 2024 (2nd intervention arm) [89] | N/A | Register on the E4T website (2-step sign-up) and purchase higher-point (healthier) food/drink options in the school canteen on most days over the approx. 4-month scheme to accumulate points; top-tier reward required the healthiest breakfast/lunch choices on most days of the intervention, lowest-tier reward required healthier purchases consistently for approx. 2 weeks | Step 1 (website sign-up) completed by 76.0% (177/233) of consenting pupils; Step 2 (full registration/account activation) completed by 44.6% (104/233); of all 85 rewards claimed across both intervention schools, 19 (22%) were claimed by pupils at this school; 12 pupils (29% of all reward-claimers across both schools) claimed at least one reward, approx. 9.7% of this school's registered pupils; of 179 registered pupils across both intervention schools combined, 35 (19.6%) downloaded the Android app (not split by school) | N/A | 44.6% | N/A |

^1^Proportion of participants who completed the entire intervention. ^2^Overall intervention adherence rate or mean adherence rate across all intervention components.

**Table S11.** Obesity: Study Characteristics.

| Authors | Country | Setting | Delivery type | Level of personal support^1^ | Incentives^2^ | Number of participants | Mean age (SD) ^3^ | Intervention duration in days |
| --- | --- | --- | --- | --- | --- | --- | --- | --- |
| Bauer et al., 2010 [47] | Netherlands | Mobile | SMS | No | None | 40 | 10.05 (1.28) | 252 |
| Bent et al., 2023 [34] | USA | Online | App | Continuous | None | 15 | 17.5 (2.2) | 91 |
| Chew et al., 2021 [42] | Singapore | Online | App | Continuous | None | 40 | 13.8 (1.7) | 84 |
| Deslippe et al., 2023 [35]   Deslippe et al., 2023 (2nd intervention arm) [35] | Canada | Online | App | Continuous | None | 82 | 10.9 (N/A) | 90 |
|  | Canada | Online | App | Continuous | None | 91 | 14.6 (N/A) | 90 |
| Doyle et al., 2008 [127] | USA | Online | Internet | Continuous | Lottery system based on task completion | 40 | 14.9 (1.7) | 112 |
| Furlong, 2021 [44] | USA | School-based | Online platform Nearpod | None | None | 136 | N/A | 14 |
| Jones et al., 2008 [37] | USA | Online | Internet | Continuous | None | 52 | 15.0 (1.0) | 112 |
| Kornman et al., 2010 [38] | Australia | Online | SMS + email | Continuous | None | 49 | 14.3 (0.9) | 365 |
| Likhitweerawong et al., 2021 [128] | Thailand | Online | App | None | None | 38 | 13.11 (1.99) | N/A |
| Lopez et al., 2021 [129] | USA | Online | App | Continuous | Gift cards | 26 | 15.39 (1.26) | 180 |
| Lopez et al., 2021 (2nd intervention arm) [129] | USA | Online | App | Continuous | Gift cards | 28 | 15.39 (1.26) | 180 |
| Lubans et al., 2014 [43] | Australia | School-based | App + Internet | Continuous | None | 361 | 12.7 (0.5) | 140 |
| Mâsse et al., 2015 [44] | Canada | Home-based | Internet | Continuous | None | 160 | 13.2 (1.8) | 224 |
| Naets et al., 2020 [48] | Belgium | Clinic-based | Internet | Continuous | None | 68 | 16 (2) | 98 |
| Stasinaki et al., 2021 [46] | Switzerland | Online | App | Continuous | Smartphone prize draw | 18 | 12.6 (11.4-16.9) | 165 |
| Tu et al., 2017 [36] | Canada | Online | Internet | Continuous | None | 159 | 13.2 (1.8) | 238 |
| Vidmar et al., 2019 [130] | USA | Online | App + SMS | Continuous | 300$ | 18 | 14.44 (± 1.65) | 182 |
| Vidmar et al., 2023 [39] | USA | Online | App | Continuous | N/A | 54 | 15 (14–17) | 182 |
| Vidmar et al., 2023 (2nd intervention arm) [39] | USA | Online | App | None | N/A | 53 | 16 (15–16) | 182 |
| (White et al., 2004) [41] | USA | Home-based | Internet | Continuous | None | 28 | 13.14 (1.59) | 180 |
| Whittemore et al., 2013a; Whittemore et al., 2013b [40] | USA | School-based | Internet | Continuous | Gift card for completion of data collection | 177 | 15.3 (0.69) | Ca.90 |
| Whittemore et al., 2013a; Whittemore et al., 2013b (2nd intervention arm) [40] | USA | School-based | Internet | Continuous | Gift card for completion of data collection | 207 | 15.3 (0.69) | Ca. 90 |

^1^Personal support categorized as: none, continuous, or not applicable (N/A); detailed descriptions provided for cases outside these categories. ^2^Monetary incentives converted to USD. ^3^Age reported as available when mean and standard deviation not provided in original article. **Table S12.** Obesity: Adherence-related Study Characteristics.

| Authors | NSessions | Intended use | Reported adherence | Rate of intervention completion^1^ | Adherence rate^2^ | Attrition |
| --- | --- | --- | --- | --- | --- | --- |
| Bauer et al., 2010 [47] | N/A | Weekly self-monitoring | On average, children stayed in the program for a duration of 32.60 weeks (SD = 9.69). During their participation, they submitted on average 67% of the expected weekly SMS (boys: 68%, girls: 66%). Ten children (25%) sent in more than 75% of the requested 36 weekly SMS. Five children (12.5%) submitted less than 25% of the requested SMS. | N/A | 67% | N/A |
| Bent et al., 2023 [34] | N/A | App usage (≥3 uses per week considered active) | Number of active app users 53% in week 1, 20% in week 2; Temporary increases in app use occurred at both the 4- and 8-week period, which correlated to the time of their virtual check-in with the athletic trainer | N/A | N/A | 33.3% |
| Chew et al., 2021 [42] | N/A | Weekly self-monitoring | During the 12-week program, participants logged mean of 4.1 (SD 2.1) weight measurements; recorded physical activity 3.2 (SD 2.6) times each week. | N/A | 34.2% | 50% |
| Deslippe et al., 2023 [35]   Deslippe et al., 2023 (2nd intervention arm) [35] | N/A | Engagement with social features | At 4 weeks, approximately 50% of preteens were still using the app; app use was 92.4 minutes on average | N/A | N/A | N/A |
|  | N/A | Engagement with social features | At 4 weeks, approximately 30% of teens were still using the app; app use was 72.2 minutes on average | N/A | N/A | N/A |
| Doyle et al., 2008 [127] | N/A | N/A | 29.9% of screens accessed on average | N/A | 29.9% | N/A |
| Furlong, 2021 [45] | 3 | Completion of 3 modules | Average engagement 70.14% | N/A | 70.14% | N/A |
| Jones et al., 2008 [37] | N/A | Completion of questionnaires and interviews | 27% of all SB2-BED participants used some component of the online program for more than or equal to 8 weeks, 42% used the program for 1 to 7 weeks, and 31% never logged on. | N/A | N/A | 11.5% |
| Kornman et al., 2010 [38] | 8 messages | Engagement in the e-contact intervention was defined by the number of replies received overall from adolescents and was classified as high (3 replies) or low (0–2 replies). | 22% reply rate overall. At least one reply was received from most (n ¼ 43) adolescents. Adolescent reply rates to individual messages ranged from 0% to 55%. | N/A | 22% | 7.1% |
| Likhitweerawong et al., 2021 [128] | N/A | N/A | The average compliance rate in using the OBEST application among participants in the intervention group was approximately 50%. This rate was measured by the frequency recording of daily food ingestion during the study period | N/A | 50% | 0% |
| Lopez et al., 2021 [129] | 24 weekly phone calls | Daily app usage | Specifically, participants assigned to the control group had the highest level of engagement (83%) compared with participants in the AppCoach (63.5%) and AppAlone (22.5%) | N/A | 63.5% | 11.5% |
| Lopez et al., 2021 (2nd intervention arm) [129] | N/A | Daily app usage | Specifically, participants assigned to the control group had the highest level of engagement (83%) compared with participants in the AppCoach (63.5%) and AppAlone (22.5%) | N/A | 22.5% | 39.3% |
| Lubans et al., 2014 [43] | N/A | N/A | 70% used goal setting function; 62% used technique function; 49% used step tracking and fitness challenge functions; Approximately, 20% of participants did not engage with the app at all | N/A | 60% | N/A |
| Mâsse et al., 2015 [44] | N/A | Weekly logins | On average, adolescents completed about 16 weeks (SD=12) of the 32-week intervention; In the first 4 months, logging in averaged 50.0 % (SD=12.2 %); Mean 38.1% of web pages viewed in first 4 months; in the last 4 months of the intervention, logging in dropped to 30.3 %; Mean 18.0% of web pages viewed in last 4 months; Tracking tools used 24.3% of weeks on average; in the first 4 months, 17 % logged in every week but 73 % logged in on some of the weeks; in the last 4 months, 6.3 % logged in every week and 50.3 % logged in on some of the weeks; they tracked their health behaviors for 24.3 % (SD=29.2) of the weeks during the study period | N/A | 11.65% | N/A |
| Naets et al., 2020 [48] | 20 | 20 sessions | 97% adherence during intensive inpatient phase; 24% adherence during home-based booster phase | N/A | 24% | N/A |
| Stasinaki et al., 2021 [46] | N/A | N/A | Average daily PM app usage rate was 71.5%; the average adherence rate was 57.2% during the first 5.5 months of the study | N/A | 57.2% | 6% |
| Tu et al., 2017 [36] | N/A | Weekly login | Mean adolescent participation rate: 28.4%; Mean participation rate in the first 4 months was 38.1% (32.3); Mean participation rate for the last 4 months was 18% (24.2) | N/A | 28.4% | N/A |
| Vidmar et al., 2019 [130] | N/A | 100% clinic visit + 100% phone session | 100% of the app intervention group attended all scheduled face-to-face visits, and 72% (13/18) participated in at least 80% of the scheduled phone meetings. Further, app participants interacted with the app 1–8 times per day (3.23 daily average) and spent 4 min 29 s daily in the app. | N/A | N/A | 0% |
| Vidmar et al., 2023 [39] | 6 | Attending 6 clinic sessions | Of the participants who attended the week 24 study visit, 54% completed all three phases of the app program; On average, participants across both app groups, engaged with the app 37% of the prescribed time (~68/182 days, range 0–182 days). | 54% | 37% | 31.5% |
| Vidmar et al., 2023 (2nd intervention arm) [39] | N/A | N/A | Of the participants who attended the week 24 study visit, 69.5% completed all three phases of the app program; On average, participants across both app groups, engaged with the app 37% of the prescribed time (~68/182 days, range 0–182 days). | 69.5% | 37% | 56.6% |
| White et al., 2004 [41] | 26 | N/A | Mean website hits in behavioral group: 624.9 (SD = 444.1); Mean completed quizzes: 9.75 (SD = 8.17); Mean completed quizzes weeks 1 to 13: 5.93 (4.15); mean completed quizzes weeks 14 to 26: 2.04 (3.91). | N/A | N/A | 17.9% |
| Whittemore et al., 2013a; Whittemore et al., 2013b [40] | 8 | 100% completion | 69.5% completed all sessions; Participation was also high, with adolescents completing 83% of lessons. | 69.5% | 83% | 5% |
| Whittemore et al., 2013a; Whittemore et al., 2013b (2nd intervention arm) [40] | 12 | 100% completion | 52.7% completed all sessions; Participation was also high, with adolescents completing 83% of lessons. | 52.7% | 83% | 3% |

^1^Proportion of participants who completed the entire intervention. ^2^Overall intervention adherence rate or mean adherence rate across all intervention components.

**Table S13.** Tobacco: Study Characteristics.

| Authors | Country | Setting | Delivery type | Personal support^1^ | Incentives^2^ | Number of participants | Mean age (SD)^3^ | Intervention duration in days |
| --- | --- | --- | --- | --- | --- | --- | --- | --- |
| Aveyard et al., 2003 [131] | UK | School-based | Computer program | Continuous | None | 4125 | 14.6 (0.3) | N/A |
| Braciszewski et al., 2024 [55] | USA | Online | Internet + SMS | N/A | Gift cards + prize draw | 28 | 19.18 (0.79) | 180 |
| Buller et al., 2008 [57]  Buller et al., 2008 (2nd intervention arm) [57] | Australia | School-based | Internet | Continuous | None | 754 | 12-16 years | N/A |
|  | USA | School-based | Internet | Continuous | None | 640 | 11-13 years | N/A |
| Chan et al., 2021 [56] | USA | Online | SMS | N/A | None | 2685 | 17.33 (1.59) | At least 42 days |
| Mason et al., 2016 [132] | USA | Online | SMS | One in-person motivational interview | None | 100 | 16.2 (1.39) | 5 |
| Mays et al., 2017 [133] | USA | Online | Internet | N/A | None | 319 | 15.0 (1.6) | 30 |
| Pallonen et al., 1998 [134]   Pallonen et al., 1998 (2nd intervention arm) [134] | USA | School-based | Computer | Continuous | None | 88 | 16.5 (0.9) | 180 |
|  | USA | School-based | Computer | Continuous | None | 47 | 16.5 (0.9) | 180 |
| Patten CA et al., 2006 [135] | USA | Home-based | Internet | Continuous | 10$ for each assessment and 20$ for last assessment | 70 | 15.7 (1.3) | 168 |
| Pbert et al., 2020 [136] | USA | Online | App | Continuous | 25$ gift cards for assessment completion | 48 | 16.9 (1.1) | 28 |
| Pbert et al., 2020 (2nd intervention arm) [136] | USA | Online | App | Continuous | 25$ gift cards for assessment completion | 50 | 17.1 (1.2) | 28 |
| Graham et al., 2024 [94] | USA | Online | Automated interactive SMS text message program (This is Quitting) | None (fully automated; no human coach) | $5 per 14-day check-in text + $20/survey (1-, 7-month) + $10 bonus for response <24h (retention incentives, same both arms) | 759 | 16.4 (0.8) | 28-98 (variable by quit-date status) |
| Ling et al., 2026 [92] | USA | Online | Instagram DM support group (counselor-facilitated) | Continuous | $10-$25 survey incentives + $5/$5 bonuses (survey-completion incentive, not intervention-specific; p. 3) | 256 | 18.89 (1.9) | 35 |
| Lustig et al., 2026 [91] | USA | Clinical | SMS outreach (opt-in question on visit questionnaire; navigator-sent texts) | Continuous (human remote health navigator) | None | 438 | 16.7 (1.72) | 63 |
| Lustig et al., 2026 (2nd intervention arm) [91] | USA | Clinical | SMS outreach (automated Twilio bot; navigator follow-up if interested) | None initially (automated bot), then continuous (human navigator) once interest expressed | None | 481 | 16.8 (1.83) | 63 |
| Mays et al., 2024 [90] | USA | Clinical | Tablet screening + in-clinic provider counseling + referral to online e-health game (ASPIRE) | Brief in-clinic provider counseling (single visit) + automated email reminders | N/A | 388 | 14.7 (1.7) | 30 |
| Sadasivam et al., 2026 [93] | USA | School-based | Multicomponent texting platform (peer messages, peer videos, peer coaching, gamified story) | Continuous (asynchronous peer coaching) | $50 (assessment completion, not intervention-contingent) | 39 | 17.3 (1.2) | 90 |

^1^Personal support categorized as: none, continuous, or not applicable (N/A); detailed descriptions provided for cases outside these categories. ^2^Monetary incentives converted to USD. ^3^Age reported as available when mean and standard deviation not provided in original article.
**Table S14.** Tobacco: Adherence-related Study Characteristics.

| Authors | NSessions | Intended use | Reported adherence | Rate of intervention completion^1^ | Adherence rate^2^ | Attrition |
| --- | --- | --- | --- | --- | --- | --- |
| Aveyard et al., 2003 [131] | 6 (3 in class, 3 on computer) | Using 2 items from mandatory questionnaire. | over 77% receiving all three computerised interventions | 77% | N/A | N/A |
| Braciszewski et al., 2024 [55] | Daily SMS | Response to two weekly questions via SMS | 73% overall response rate to weekly poll questions for intervention group | N/A | 73% | 28.6% |
| Buller et al., 2008 [57] | 73 online activites in 6 modules | Completion of activities in 6 modules | Response rate of 81.2%; 91% of children completed Module 1 (only 6% did not use Module 1). Completion of subsequent modules decreased (54.6% Module 2, 57.7% Module 3, 45.5% Module 4, 33.2% Module 5, 24.6% Module 6). On average, children completed 43.2 out of 73 activities (59%); Only 26.0% of students completed at least 90% of activities and could be reasonably considered to have completed the program. | N/A | 59% | 28.8% |
| Buller et al., 2008 (2nd intervention arm) [57] | 73 online activities in 6 modules | Completion of 6 modules | 83.1% of children completed Module 1, but fewer completed subsequent modules (47.5% Module 2, 45.6% Module 3, 42.0% Module 4, 42.0% Module 5, 18.6% Module 6). On average, children completed 46.6 out of 73.0 activities (64%) in Consider This, but only 24.8% of students completed at least 90% of activities and could be reasonably considered to have completed the program. | N/A | 64% | 16.9% |
| Chan et al., 2021 [56] | N/A | N/A | Among noncompleters (n = 1,733, 64.54%), 27.69% dropped out prior to their quit day and 72.27% dropped out on or after quit day (Table 1). Time from quit day to dropout averaged 8 days. The survival rate was around 45% for nondaily and daily smokers by intervention end (Day 42; Fig. 1). | 45.46% | N/A | 64.5% |
| Mason et al., 2016 [132] | 30 SMS | 100% response to SMS + 100% requesting booster sessions | Participants in Experimental condition completed 80.5 % of all responses to texting intervention. Nearly one-fifth (19 %) of the adolescents in the experimental condition requested booster messages, with a mean of 2.3 boosts per teen (SD = 1) | N/A | 80.5% | 13% |
| Mays et al., 2017 [133] | N/A | Website engagement | 25.9% pf participants overall reported engaging with the website; 60.6% visited the site once; 22.5% twice, 4.2% three times and 12.7% four or more times | N/A | N/A | 12.5% |
| Pallonen et al., 1998 [134] | 3 | Completion of sessions | 62.5% completed all session; 26.1% completed two sessions; 11.4% completed 1 session | 62.5% | N/A | 42% |
| Pallonen et al., 1998 (2nd intervention arm) [134] | 3 | Completion of sessions | 74.5% completed all sessions; 12.8% completed two sessions; 12.7% completed one session | 74.5% | N/A | 42% |
| Patten CA et al., 2006 [135] | N/A | N/A | By the end of week 3, 86% had used the SOS site at least once; Use dropped to less than one-third of participants by week 3; mean number of days of use for all SOS participants was 6.8; percentage of SOS users decreased significantly over the 24 weeks | N/A | 4% | N/A |
| Pbert et al., 2020 [136] | 22 | N/A | Average 61% modules completed; 38% completed all 22 modules | 38% | 61% | 4% |
| Pbert et al., 2020 (2nd intervention arm) [136] | N/A | N/A | Using the app a total of 12.3 days on average (range 0-62) over the intervention period. | N/A | 43.9% | 0% |
| Graham et al., 2024 [94] | N/A | N/A (ongoing automated program; no discrete session target; users may set/reset a quit date and access on-demand support via keywords TIPS/FEELS/STRESS, p. 3) | Not reported: no engagement/usage metrics (e.g., messages sent/received, quit-date setting rate) were reported for the SMS program itself; only survey follow-up/retention rates were reported | N/A | N/A | 19.76% (150/759 lost to follow-up at 1-month assessment, used as proxy for during-intervention attrition) |
| Ling et al., 2026 [92] | N/A | N/A | 88% (104/121 of 5-week survey completers) reported reading chats at least once a day (36% daily, 52% more than once a day); 85% (103/121) reported posting or commenting at least once a week; 41% posted at least once a day; 59.7% posted/commented on the last day of the group; 44% posted/liked at least 25 times over the 25-day intervention; 89% talked to others in the past month about the program | N/A | N/A | 51.6% |
| Lustig et al., 2026 [91] | N/A | N/A | 90% provided a phone number for outreach (exact Phase 1 n not separately reported, only phase-level %); 11% (50/438) expressed interest in cessation resources; 2% (10/438) connected to treatment (TIQ enrollment and/or NRT prescription); at 2-week follow-up, 1% (6/438) reported using NRT and/or participating in TIQ; 1 of the 10 treatment-connected patients was lost to follow-up after connecting to TIQ | N/A | N/A | N/A |
| Lustig et al., 2026 (2nd intervention arm) [91] | N/A | N/A | 91% provided a phone number for outreach (exact Phase 2 n not separately reported, only phase-level %); 45% (194/428) of Twilio-generated outreach texts were automatically blocked as spam by cell carriers; 2% (9/483) expressed interest in cessation resources; 1% (6/481) connected to treatment; at 2-week follow-up, 0.5% (3/481) reported using NRT and/or participating in TIQ | N/A | N/A | N/A |
| Mays et al., 2024 [90] | N/A | N/A | 80.9% (314/388) engaged with ASPIRE at least once; 63.1% (245/388) engaged at first prompt; 17.8% (69/388) engaged only after multiple prompts; 19.1% (74/388) did not engage at all | N/A | 80.9% | 11.6% |
| Sadasivam et al., 2026 [93] | N/A | Daily peer messages days 1-30; 2-3x/week peer messages months 2-3; peer videos sent at intervals during days 1-30; gamified story installment daily (auto-advances after 3 days without reply); peer coach available for participant-initiated contact throughout 3 months, coach to respond within 24-48h | High engagement (self-reported use always/usually/about half the time): peer messaging 80.6% (29/36); gamification 50.0% (18/36); peer coaching 48.7% (18/37); peer videos 37.1% (13/35); Engagement Summary Score (range 0-4) mean 2.1 (SD 1.4); peer coach contact frequency range 0-17 per participant; Satisfaction Summary Score (range 8-32) mean 25.3 (SD 4.7) | N/A | 54.1% (derived mean of the four component high-engagement rates above; not reported directly by the study) | 5.1% |

^1^Proportion of participants who completed the entire intervention. ^2^Overall intervention adherence rate or mean adherence rate across all intervention components.

**Table S15.** Alcohol: Study Characteristics.

| Authors | Country | Setting | Delivery type | Level of personal support^1^ | Incentives^2^ | Number of participants | Mean age (SD) ^3^ | Intervention duration^4^ |
| --- | --- | --- | --- | --- | --- | --- | --- | --- |
| Haug et al., 2017 [58] | Switzerland | Online | Web + SMS | N/A | 11$ | 547 | 16.9 (1.6) | 84 |
| Ho et al., 2021 [137] | Hong-Kong | Online | Internet | N/A | Win prizes | 4294 | 13.15 (1.17) | 30 |
| Jander et al., 2016 [60] | Netherlands | School-based | Computer | N/A | None | 1622 | 16.0 (1.2) | 120 |
| Martinez-Montilla et al., 2020 [59] | Spain | School-based and mobile | Internet | Continuous | None | 742 | 16.40 (1.06) | N/A |
| Newton et al. 2022 [138] | Australia | School-based | Internet | Continuous | None | 3236 | 13.5 (0.6) | 180 |
| Schinke et al., 2010 [139] | USA | Community-based | CD-Rom | One arm of the intervention group had parental support | Giftcards | 188 | 10.79 (1.0) | 210 |
| Vargas-Martínez et al., 2019 [140] | Spain | Online | Internet | N/A | None | 742 | 16.8 (0.02) | 120 |
| Fernández-León et al., 2026 [95] | Spain | School-based | Web-based CT game (animation + gamification), computer-delivered in classroom | Continuous | None | 35 | 16.57 (1.008) | 2 |
| Wall et al., 2024 [96] | Sweden | Online | Website (3 film-based lectures + automated feedback + 4 vignette exercises + individualized therapist feedback) | Therapist-assisted (individualized written feedback) | US$12 per completed assessment (up to US$48 total) | 101 | 17.0 (1.29) | N/A |
| Wang-Schweig et al., 2025 (parent participants) [97] | USA | Online | Web-based family (parent-teen dyad) prevention program: video narratives, info-gadgets, interactive choice-based activities, offline discussion guides; 3 components (Communications, Alcohol, Relationships) | Continuous (biweekly research-staff phone calls until completion or 6 months; text reminder after 10 days no-login; email reminder after 14 days; in-app parent/teen 'nudge' feature) | $50 weekly lottery per completing family | 206 | 43.7 (6.7) | N/A |
| Wang-Schweig et al., 2025 (teen participants) [97] | USA | Online | Web-based family (parent-teen dyad) prevention program: video narratives, info-gadgets, interactive choice-based activities, offline discussion guides; 3 components (Communications, Alcohol, Relationships) | Continuous (biweekly research-staff phone calls until completion or 6 months; text reminder after 10 days no-login; email reminder after 14 days; in-app parent/teen 'nudge' feature) | $50 weekly lottery per completing family | 206 | 16.4 (0.5) | N/A |

^1^Personal support categorized as: none, continuous, or not applicable (N/A); detailed descriptions provided for cases outside these categories. ^2^Monetary incentives converted to USD. ^3^Age reported as available when mean and standard deviation not provided in original article.
**Table S16.** Alcohol: Adherence-related Study Characteristics.

| Authors | NSessions | Intended use | Reported adherence | Rate of intervention completion^1^ | Adherence rate^2^ | Attrition |
| --- | --- | --- | --- | --- | --- | --- |
| Haug et al., 2017 [58] | N/A | N/A | 65.6% read the SMS messages thoroughly, 32.6% took a quick look at the SMS messages, and 1.7% did not read the SMS messages | N/A | N/A | 6.6% |
| Ho et al., 2021 [137] | N/A | Access the website | 46% of participants accessed website | 46% | N/A | 16% |
| Jander et al., 2016 [60] | 5 | Attend 5 sessions | 67.6% started with the first game session, 28.8% returned to the second, and 21.4% adolescents to the third game session. 1.7% adolescents returned to the fourth session at home, and zero participated in the fifth home session. | 0% | 23.9% | 71.9% |
| Martinez-Montilla et al., 2020 [59] | 6 | Completion of 6 sessions | 62.1% started the second session at school, 47.2% returned for the third session at school, 3.1% returned for the fourth session at home and 1.1% returned for the fifth session at home. 48.2% completed the 6^th^ session (which was the posttest) | N/A | 28.4% (only sessions 2-4) | 52.7% |
| Newton et al. 2022 [138] | 12 | Completion of 12 sessions | Completion rates for each of the first 6 sessions ranged from 97% to 98% and for the remaining six lesson from 94% to 97%. | N/A | 94-97% | 4.8% |
| Schinke et al., 2010 [139] | 10 | Completion of 10 sessions | Roughly 90% of youths assigned to the intervention arm completed the entire initial 10-session prevention program. Nearly 80% completed at least three of the five booster sessions, with 56% completing four or more booster sessions. | 90% | N/A | N/A |
| Vargas-Martínez et al., 2019 [140] | 6 | Completion of 6 sessions | Average of 2.604 (SD 1.24) sessions completed for the intervention group. | N/A | 43.4% | 53% |
| Fernández-León et al., 2026 [95] | 5 | Complete all 5 sessions (~1 hour each) across 2 school days, supervised in class by a teacher/researcher | 35/35 participants completed the five-session program; "no missing data" reported for the self-report questionnaire; all outcome measures collected immediately after Session 5 | 100% | N/A | 0% |
| Wall et al., 2024 [96] | 15 | Complete all 15 sequential program components (incl. required breaks); ~1 hour total active content | 63% (64/101) registered an account; 35% (35/101) completed the alcohol intervention section; 24% (24/101) completed both alcohol and coping sections; 37% (37/101) never logged in at all; no dose-response relationship between amount completed and outcome | 24% | N/A | N/A |
| Wang-Schweig et al., 2025 (parent participants) [97] | 3 | Both parent and teen must complete each of 3 components (Communications, Alcohol, Relationships) plus the linked offline discussion guide, in sequence, before advancing, then complete a final survey, to count as a family completer | 84% of 206 families initiated (both parent and teen logged in); among n=173 families categorized for retention: 35.4% did not complete a component, 9.2% completed 1-2 components, 39.3% completed all 3; parents in completing families used the in-app nudge feature far more than non-completers (M=5.07 nudges, SD=1.97 vs M=2.03, SD=1.56; t(139.60)=10.27, p<.001); parents' mean time spent across the 3 components M=70.40 min (SD=69.05), most time in Communications M=28.12 min (SD=22.39) | 39.3% | N/A | 60.7% |
| Wang-Schweig et al., 2025 (teen participants) [97] | 3 | Both parent and teen must complete each of 3 components (Communications, Alcohol, Relationships) plus the linked offline discussion guide, in sequence, before advancing, then complete a final survey, to count as a family completer | 84% of 206 families initiated (both parent and teen logged in); among n=173 families categorized for retention: 35.4% did not complete a component, 9.2% completed 1-2 components, 39.3% completed all 3; teens in completing families used the in-app nudge feature far more than non-completers (M=3.99 nudges, SD=1.55 vs M=1.80, SD=1.11; t(115)=7.56, p<.001); teens' mean time spent across the 3 components M=66.60 min (SD=71.42), most time in Relationships M=24.44 min (SD=35.07) | 39.3% | N/A | 60.7% |

^1^Proportion of participants who completed the entire intervention. ^2^Overall intervention adherence rate or mean adherence rate across all intervention components.

**Table S17.** Multicomponent Lifestyle Interventions: Study Characteristics.

| Authors | Country | Health domain | Setting | Delivery type | Level of personal support^1^ | Incentives^2^ | Number of participants | Mean age (SD)^3^ | Intervention duration in days |
| --- | --- | --- | --- | --- | --- | --- | --- | --- | --- |
| Abroms et al., 2004 [141] | USA | Tobacco + Diet + PA | Online | E-Mail | None | None | 37 | 15.7 (N/A) | 210 |
| Balsa et al., 2014 [63] | Uruguay | Alcohol + Tobacco | Online | Internet | Continuous | None | 359 | 15.4 (N/A) | 93 |
| Brannon et al., 2016 [64] | USA | Diet + PA | Online | App + accelometer + physical monitoring system | None | 40$ | 20 | 15.67 (1.75) | 20 |
| Cameron et al., 2015 [73] | UK | PA + Diet + Alcohol + Tobacco | Online | Internet | None | 133$ prize draw | 1346 | 18.73 (2.01) | N/A |
| Castro et al., 2017 [72] | Switzerland | Alcohol + Tobacco | Online | SMS + Internet | None | 11$ | 1418 | 18.6 (3.1) | 91 |
| DeBar et al., 2009 [66] | USA | Diet + PA | Online | Internet | Continuous | Incentives for local merchants | 82 | 15.6 (0.6) | 730 |
| Egilsson et al., 2023 [74] | Iceland | Diet + PA | Online | App | None | Altruistic rewards | 117 | 13.5 (0.63) | 42 |
| Epton et al., 2014 [75] | UK | PA + Diet + Alcohol + Tobacco | Online | App + Website | None | 13$ + 100$ prize draw | 736 | 18.76 (1.99) | 180 |
| Fuemmeler et al., 2020 [65] | USA | Diet + PA | Home-based | App + Pedometer | Continuous | 100$ | 15 | 14.8 (1.97) | 60 |
| Haug et al., 2021; Paz Castro et al., 2022a; Paz Castro et al., 2022b [69] | Switzerland | Alcohol + Tobacco | Online | SMS + Internet | None | 11$ + prize draw | 750 | 15.4 (1.0) | 154 |
| Kramer et al., 2018 [67] | USA | Obesity + PA + Diet | Summer Camp + online | In-person camp + internet | Continuous | None | 21 | 11.3 (0.97) | 63 |
| Kramer et al., 2018 (2nd intervention arm) [67] | USA | Obesity + PA + Diet | Summer camp + online | In-person camp + internet | Continuous | None | 20 | 12.4 (1.47) | 63 |
| Lin & Mâsse, 2021 [61] | Canada | Diet + PA | N/A | Internet and/or app | Continuous | None | 301 | 14.83 (1.43) | 140 |
| Maenhout et al., 2022 [62] | Belgium | Diet + PA | Online | App + accelometer | None | Powerbank + Cinema tickets | 186 | 13.51 (0.96) | 85 |
| Majumdar et al., 2013 [142] | USA | Diet + PA | N/A | Game engine | None | None | 359 | 11.4 (N/A) | 30 |
| Rydell et al., 2005 [68] | USA | Diet + PA | Community-based | Internet | Continuous | Small prizes | 194 | 10-12 years | 730 |
| Schwinn et al., 2018 [71] | USA | Alcohol + Tobacco | Online | Internet | Reminders | 90$ | 396 | 13.66 (0.67) | 98 |
| Schwinn et al., 2021 [143] | USA | Alcohol + Tobacco | Online | App | None | Gift cards | 321 | 14.16 (1.12) | 135 on average |
| Spook et al., 2016 [76] | Netherlands | Diet + PA | Online | App + Website | None | Win 22$ vouchers | 228 | 16.96 (1.1) | 28 |
| Audi et al., 2026 [101] | Brazil | Obesity + Diet | Clinical | App (FatSecret) for daily dietary self-monitoring + individualized energy-restricted diet | Continuous (monthly clinic visits + biweekly WhatsApp/email/phone contact) | None | 30 | 15 (N/A) | 168 |
| Berdiyarova et al., 2026 [98] | Uzbekistan | Diet + PA + Obesity | Clinical (recruitment and monthly counseling at a pediatric medical center; app used at home) | Gamified app ("PressPlay Kids": Food Explorer, Step Quest, Pill Pal modules) + standard counseling | Minimal/automated - same monthly 30-min nutritionist session as control; weekly automated SMS summary to parents; no continuous human coaching within the app itself | None | 40 | 12.7 (1.9) | 84 |
| Bjerregaard et al., 2024 [105] | Denmark | Diet + Obesity | Online/remote (SMS to personal mobile phone; no institutional site) | SMS chatbot educational program (automated two-way text messaging) | None | Non-monetary (competition prizes: gift cards to amusement parks/shops, cinema tickets; no fixed USD amount stated) | 5260 | N/A (not reported) | N/A (mixed: 84 days FULL program / 28 days targeted program) |
| Chen et al., 2024 [99] | Taiwan | Obesity + PA + Diet | Home-based (recruited via junior/senior high schools) | mHealth app + smartwatch (Fitbit Versa) | None (automated app-based education and reminders only) | Raffle (value not reported) | 27 | 16.32 (1.26) | 84 |
| Egan et al., 2024; O'Dean et al., 2024; O'Dean et al., 2025 [110] | Australia | Diet + PA + Alcohol + Tobacco | School-based | 6 online video/cartoon modules (in-class, ~weekly) + optional companion smartphone app (out-of-class) | None (self-directed; optional teacher-facilitated in-class activities) | None/N/A | 3609 | 12.66 (0.50) | 42 |
| Henry et al., 2025 [104] | USA | Diet + PA | Clinical (baseline/3-/4-month assessment visits); app used independently at home | App (CommitFit) - gamification + behavioral economics | None | None | 10 | 14.4 (0.84) | 120 |
| Henry et al., 2025 (2nd intervention arm) [104] | USA | Diet + PA | Clinical (baseline/3-/4-month assessment visits); app used independently at home | App (CommitFit) + financial incentive ($0.05/point, first 3 months) | None | $0.05 per point earned in-app, first 3 months only | 10 | 14.2 (1.39) | 120 |
| Mateo-Orcajada et al., 2024 [106] | Spain | PA + Obesity | School-based (app used out-of-school hours) | Step-tracker app (STRAVA) | None | None | 30 | 14.11 (1.24) | 70 |
| Oei et al., 2024 [100] | Canada | Diet + Obesity | Clinical | App (image-recognition dietary photo log) + Clinician Portal | Continuous (dietitian visits + weekly in-app tips + automated reminders) | N/A | 16 | 15.9 (1.7) | 28 |
| Parker et al., 2024 [144] | USA | Diet + Obesity | Home-based | Smartphone app (food-cue attention retraining / visual-probe task) | None | N/A | 32 | 15.00 (1.61) | 14 |
| Parker et al., 2024 (2nd intervention arm) | USA | Diet + Obesity | Home-based | Smartphone app (control/sham visual-probe task, same schedule and dose as AR) | None | N/A | 36 | 14.88 (1.70) | 14 |
| Ponce-Ramírez et al., 2024 [108] | Spain | PA + Obesity | School-based (promoted via PE class); app used out-of-school hours | App (gamified location-based game - Pokémon Go) | Minimal (researcher recorded steps only) | 10% increase in final PE-subject grade (non-monetary) | 22 | 14.25 (1.23) | 70 |
| Ponce-Ramírez et al., 2024 (2nd intervention arm) [108] | Spain | PA + Obesity | School-based (promoted via PE class); app used out-of-school hours | App (pedometer with messages/reminders - Strava) | Minimal (researcher recorded steps only) | 10% increase in final PE-subject grade (non-monetary) | 22 | 14.25 (1.23) | 70 |
| Ponce-Ramírez et al., 2024 (3rd intervention arm) [108] | Spain | PA + Obesity | School-based (promoted via PE class); app used out-of-school hours | App (pedometer with messages/reminders - Pacer) | Minimal (researcher recorded steps only) | 10% increase in final PE-subject grade (non-monetary) | 22 | 14.25 (1.23) | 70 |
| Ponce-Ramírez et al., 2024 (4th intervention arm) [108] | Spain | PA + Obesity | School-based (promoted via PE class); app used out-of-school hours | App (pedometer with messages/reminders - Map My Walk) | Minimal (researcher recorded steps only) | 10% increase in final PE-subject grade (non-monetary) | 22 | 14.25 (1.23) | 70 |
| Ramalho et al., 2025 [102] | Portugal | Diet + PA | Clinical (public hospital adjunct to treatment as usual; intervention delivered online via Facebook) | Facebook private group (CBT psychoeducational videos + weekly tasks) + self-monitoring web app with automatic feedback + Facebook Messenger chat sessions | Low/on-request (monthly 20-30 min chat sessions with an MSc psychologist, available on request, not continuous) | N/A (not reported) | 69 | 14.97 (1.68) [completer subsample n=38; full randomised-arm age N/A] | 182 |
| Roche et al., 2024 [107] | USA | Diet + PA | Online | Video series (5 modules, animated education + athlete/role-model interviews) | None (automated reminder emails only) | $30 gift card | 45 | 16.0 (1.4) | 7 |
| Roche et al., 2024 (2nd intervention arm) [107] | USA | Diet + PA | Online | Online pamphlets/readings/blogs (5 modules, same topics) | None (automated reminder emails only) | $30 gift card | 39 | 15.7 (1.0) | 7 |
| Seiterö et al., 2025 [109] | Sweden | Alcohol + Diet + PA + Tobacco | Online | App dashboard (4 modules) + weekly SMS screening + optional SMS text-message program | None (fully automated) | None | 377 | 17.1 (1.2) | 112 |

^1^Personal support categorized as: none, continuous, or not applicable (N/A); detailed descriptions provided for cases outside these categories. ^2^Monetary incentives converted to USD. ^3^Age reported as available when mean and standard deviation not provided in original article.

**Table S18.** Multicomponent Lifestyle Interventions: Adherence-related Study Characteristics.

| Authors | NSessions | Intended use | Reported adherence | Rate of intervention completion^1^ | Adherence rate^2^ | Attrition |
| --- | --- | --- | --- | --- | --- | --- |
| Abroms et al., 2004 [141] | 10 | Reply rate to quizzes / questions | On average, 36.6% of participants replied to each Ezine volume by providing answers to quiz questions or submitting advice column questions. Furthermore, almost half of the Ezine group (45.9%) replied to the Ezine at least once during the course of the intervention. | 36.6% | N/A | N/A |
| Balsa et al., 2014 [63] | N/A | Optional use of website | 21% logged in at least once; 55% logged in just once, 18% did it on two days and the remaining 27% did it on at least three days; Most visitors simply browsed the site and read posts or materials uploaded. 7% showed a stronger interest in the site and engaged in other activities, such as forums, chats, or online surveys; rate of participation as measured by the ratio of student logins per day to the total amount of student-day observations is only 0.6% | N/A | 0.6% | N/A |
| Brannon et al., 2016 [64] | 80 | 80 observations (survey completion) | Survey compliance: 81.02% | N/A | 81.02% | 10% |
| Cameron et al., 2015 [73] | 4 | Completion of tasks and modules | 85% completed the self-affirmation task; engagement with the health messages: 72 % viewed a message for at least one behaviour, 50% for at least two behaviours, 48 % for at least three behaviours, and 47% for all four behaviours; Considering engagement with the planning tasks, 41% formed an implementation intention for at least one behaviour, 36% for at least two behaviours, 33% for at least three behaviours, and 29% for all four behaviours. | N/A | N/A | 44.4% |
| Castro et al., 2017 [72] | 11 Text Messages | 11 | A mean of 6.6 (SD 3.5) out of 11 text message prompts = 60% // 20.5% (SD 31.5) of the received media content | N/A | 60% (and 20.5%) | 1.8% participants in the MCT choosing to unsubscribe compared with 2.5% dropping out of MCT+ |
| DeBar et al., 2009 [66] | N/A | N/A | Average of 23.2 website visits over 2 years; 112.1 minutes website use on average; mean of 4.4 minutes per week on study website; 56.1% used website in at least half of the study periods | N/A | N/A | N/A |
| Egilsson et al., 2023 [74] | N/A | Downloading app + completing at least 3 tasks | Mean usage duration: 24.975 days in intervention group vs 6.286 days in TAU; 55.6% completion rate; number of usage days 59.5%; | 55.6% | N/A | 44.4% |
| Epton et al., 2014 [75] | 3 | Completion of tasks | At 6-month follow-up 52% had completed the self-affirmation task, 35% had accessed the health messages, 1% had made a plan and 2% downloaded the app. | N/A | 29.33% | 23% |
| Fuemmeler et al., 2020 [65] | N/A | Log foods 2-3 times a day | 78.3% dietary data entries; 63.6% physical activity data entries; 14.5% completed challenges; 143 message postings | N/A | 52.1% | 6.25% |
| Haug et al., 2021; Paz Castro et al., 2022a; Paz Castro et al., 2022b [69] | 50 interactions | 50 | The mean number of activities carried out by participants was 23.6 (SD 15.9) / 47.2%. 9.6% of participants did not take part in any activities. Low engagement was established for 19.9% students, who interacted with it only 1 to 10 times; 14.0% of the participants interacted 11 to 20 times, 13.3% interacted 21 to 30 times, 24.9% interacted 31 to 40 times, and 18.3% interacted 41 to 50 times. | N/A | 47.2% | 10.4% |
| Kramer et al., 2018 [67] | 8 | Completion of 8 modules | The introductory period received the greatest magnitude of online traffic (107 views; 24 visitors), followed by the lessons delivered in Week 1 (86 views; 13 visitors (61.9%)), Week 2 (49 views, 14 visitors (66.6%)), and Week 8 (47 views; 13 visitors (61.9%)) (Figure 2). Overall, viewership of, and participation in, the eHealth program started high and dropped to a plateau throughout the duration of the eight-week curriculum | N/A | N/A | 47.6% |
| Kramer et al., 2018 (2nd intervention arm) [67] | 8 | Completion of 8 modules | Number of views per lesson: lesson 1) 86 views, 2) 49 views, 3) 41 views, 4) 37 views, 5) 29 views, 6) 24 views, 7) 30 views, 8) 47 views | N/A | N/A | 50% |
| Lin & Mâsse, 2021 [61] | N/A | N/A | Class 1 (31.9%) labelled as the Uninvolved regroups teens who do not use most app features. Class 2 (25.3%) labelled as the Dabblers regroups teens who minimally used all app features. Class 3 (23.9%) labelled as the Engaged regroups teens who had high use of the app features. Finally, class 4 (18.9%) labelled as the Keeners had much higher level of engagement with all app features; mean app engagement for teens in minutes 81.65 (155.15); 46.84% low engagement; 30.90% moderate engagement; 22.26% high engagement | N/A | N/A | N/A |
| Maenhout et al., 2022 [62] | N/A | N/A | 4.3% completion rate; 95.7% stopped using the app before the end of the 12 weeks; median survival time was 10 days; 30.1% only opened the app on installation day; 44.1% stopped using the app in the first week; 83% participation rate; most of the adolescents hardly spent any time in the app, with a median of 1.41 minutes per week for the self-regulation component, 0.03 minutes per week for the narrative, and 0.39 minutes per week for the chatbot; | 4.3% | N/A | 95.7% |
| Majumdar et al., 2013 [142] | 7 | Completion of all game activities | 64% completed all four game levels; 15.9% completed 3 levels; 15.9% completed 2 levels and 3.6% completed one level | 64% | N/A | 0.6% |
| Rydell et al., 2005 [68] | N/A | N/A | 82% of 5th graders and 56% of 6th graders accessed the site at least once; 48% of 5th graders and 23% of 6th graders accessed more than once; 51.3% of 5th graders and 12.9% of 6th graders posted to bulletin board; 60.5% of 5th graders completed at least one quiz; 26.4% of 6th graders completed at least one Puzzled Patty activity; 65.1% read at least one bulletin board message in 5th grad, in 6th grade it was 31.8% | N/A | N/A | N/A |
| Schwinn et al., 2018 [71] | 9 | Completion of 9 sessions | The 9-session intervention program was completed by 87% of girls | 87% | N/A | N/A |
| Schwinn et al., 2021 [143] | 10 | Completion of 10 sessions | 95% completed 1 session; 86% completed 3 sessions; 78% completed 6 sessions; 68% completed all 10 sessions | 68% | N/A | 19.5% |
| Spook et al., 2016 [76] | N/A | N/A | 27.6% of the intervention group reported using the intervention as intended | 27.6% | N/A | 53.9% |
| Audi et al., 2026 [101] | N/A | Daily dietary self-monitoring via FatSecret app; >40% of days logged considered satisfactory self-monitoring | Adherence to dietary self-monitoring ranged from 26.7% to 53.3% across follow-up assessments in the APP group (vs 43.3%-70% in CON), not significantly different between groups (P=0.681, p. 3); extent of ZBMI reduction did not vary meaningfully across adherence levels (P=0.121, p. 3) | 60% | N/A | 40% |
| Berdiyarova et al., 2026 [98] | N/A (3 gamified modules: Food Explorer, Step Quest, Pill Pal; no fixed session count specified) | Daily app engagement for 12 weeks | average 5.8 ± 1.6 logins/week; task completion rate 84% [IQR 75-90]; "Food Explorer" used 6.2 ± 2.1 times/week; "Step Quest" goals met 3.5 ± 1.4/week; "Pill Pal" streak median 28 days [IQR 14-65] (Table 6, p. 5); 86.8% rated the app "fun" or "very fun", 81.6% would recommend it to a friend (Table 7, p. 5) | 95% | 84% | 5% |
| Bjerregaard et al., 2024 [105] | N/A (not session-based; FULL program = 188 total quiz/tip/reminder messages over 12 weeks: 23 SSB, 26 FV, 24 fish, 19 physical activity, 3 fibre, 20 meal-related; each 4-week targeted program used a subset of these, p. 8) | Not specified as a discrete minimum threshold; messages sent to, and replies expected from, participants on an almost-daily basis for the duration of the program | 63% (3,338/5,260) of adolescents offered any SMS program joined it by texting 'JOIN'; 37% (1,922/5,260) never joined; among adolescents randomised to invite their mother, 49.0% (1,443/2,630) had the mother actually join; among those randomised to invite a friend, only 20.0% (517/2,630) had a friend join | N/A (not reported) | N/A (not reported; closest available metric is the 63% join/uptake rate, p. 12) | Not measured during the 4- to 12-week active intervention period itself. Loss to follow-up on the outcome questionnaires was 27.5% (1,446/5,260) at 6 months and 32.8% (1,723/5,260) at 18 months among SMS-assigned adolescents, but this occurs well after the intervention has ended, not during it. |
| Chen et al., 2024 [99] | N/A | Wear smartwatch (Fitbit Versa) continuously day and night for at least 3 months/12 weeks; complete mHealth app questionnaires (Weight Management Health Behavior Scale, Dietary Diary Scale, Health Literacy Weight Management Scale) | All 27 participants answered the questionnaire and synchronized smartwatch data; forced completion + reminder mechanism meant no missing questionnaire data; 38-item questionnaire completed in 5-7 min, 27-item dietary diary in 1-2 min; all participants found the questionnaire/app easy, but the majority found smartwatch setup/use difficult and time-consuming; all 27 completed focus groups | 100% (inferred; not explicitly stated as a completion rate) | N/A | 0% (inferred; not explicitly reported as attrition) |
| Egan et al., 2024; O'Dean et al., 2024; O'Dean et al., 2025 [110] | 6 | Complete all 6 in-class modules; optional companion app usable through 24-month follow-up | 62.1% (1960/3157 with completion data) completed all 6 lessons (O'Dean 2025); 11.3% (407/3609-3610) accessed the optional companion app, 0.1% (5) accessed selective booster content (O'Dean 2024, O'Dean 2025); nested process-evaluation subsample (Egan 2024, 6 schools/364 students) reported teacher-logged module-level engagement 85.7-100% (mean ~92%) | 62.1% | N/A | 16.0% |
| Henry et al., 2025 [104] | N/A | N/A (not explicitly specified as a minimum; app allowed up to 2 daily notification reminders, users selected 1-2 of 5 health behaviors to log over self-selected 1-4 week goal periods, p. 4) | Pooled across CommitFit and CommitFit$ (n=20 dyads; not reported separately by arm, p. 6): intervention adolescents logged in 234 times total vs. caregivers 153 times; adolescents 6/20 (30%) high loggers (>=57% logging rate), 11/20 (55%) moderate loggers (28-56%), 3/20 (15%) low loggers (<28%) (Table 3, p. 9); caregiver-adolescent logging tier positively correlated (r=0.65, P=0.002, p. 6); arm-specific gamification survey means (Table 2, p. 8): 'CommitFit had positive impact on health behavior' 55.2 vs. 73.0 (P=0.10); 'leaderboard competition motivated logging' 48.7 vs. 75.5 (P=0.04*); 'team competition with caregiver motivated logging' 40.5 vs. 63.1 (P=0.10); 'liking of caregiver also using app/competing on family leaderboard' 68.1 vs. 75.6 (P=0.46) | 100% | N/A | 0% |
| Henry et al., 2025 (2nd intervention arm) [104] | N/A | N/A (same as above, p. 4) | Pooled across CommitFit and CommitFit$ (n=20 dyads; not reported separately by arm, p. 6) - see identical figures in the CommitFit row above (Table 3 p. 9; Table 2 p. 8, CommitFit$ column values: 73.0 / 75.5* / 63.1 / 75.6) | 90% | N/A | 10% |
| Mateo-Orcajada et al., 2024 [106] | N/A | Use STRAVA app min. 3x/week out-of-school; walk incrementally increasing distance from 4600 steps/day (week 1) to 10,000 steps/day (week 10); cumulative target 139.97 km over 10 weeks | Average distance traveled 76.44 km (SD 39.10) over 10 weeks (range 14.43-186.2 km); only 12% (3/26) reached/exceeded the 139.97 km target; 54% (14/26) stopped systematic app use after week 6 | 86.7% | 12% | 13.33% |
| Oei et al., 2024 [100] | N/A | Take photos of all meals/snacks/beverages daily during 4-week study; target >=80% completion of food documenting/photo-taking (implementation criterion, p. 4) | mean 1.7 (SD 1.9) photos/day (experimental) vs 0.9 (SD 0.7) photos/day (control), P=.14; 1 outlier took 195 photos (mean 6.1/day); engagement categories among 15 completers: no/little n=4 (<10 photos), moderate n=6 (10-45 photos), high n=5 (>45 photos), high-engagement each >=79 photos with 80% (4/5) >100 photos; technical difficulties: missed push notifications 10/15 (67%), missed dietitian tips 11/15 (73%), app crashes 5/15 (33%), deleted photos 3/15 (20%); AES acceptability mean 4.1 (SD 0.7)/5; 93% (13/14) preferred E4W over traditional logging | 94% | N/A | 6.25% |
| Parker et al., 2024 [144] | 42 (3/day x 14 days; plus 1 daily AB assessment not part of intervention dose) | Complete 3 trainings/day (80 trials each) for 2 weeks | All participants who remained in the study were reported to have completed the assigned smartphone program with no adverse events during the program itself; however CONSORT diagram shows 2/32 AR participants listed as 'did not complete'; per-training completion percentages not reported | 93.75% | N/A | 6.25% |
| Parker et al., 2024 (2nd intervention arm) [144] | 42 (3/day x 14 days; plus 1 daily AB assessment not part of intervention dose) | Complete 3 trainings/day (80 trials each) for 2 weeks | Same program structure and reporting as AR arm; CONSORT diagram shows 5/36 Control participants listed as 'did not complete'; per-training completion percentages not reported | 86.11% | N/A | 13.89% |
| Ponce-Ramírez et al., 2024 [108] | N/A | Use app minimum 3x/week; progressive daily step target 7152→12520 steps over 10 weeks | 21/22 (95.45%) completed the 10-week intervention; 1/22 (4.55%) dropout; adjusted residual for dropout/nondropout = 2.1/-2.1, near the ±1.96 significance threshold; omnibus chi-square across apps not significant (χ2=4.75, p=0.191) | 95.45% | N/A | 4.55% |
| Ponce-Ramírez et al., 2024 (2nd intervention arm) [108] | N/A | Use app minimum 3x/week; progressive daily step target 7152→12520 steps over 10 weeks | 17/22 (77.27%) completed; 5/22 (22.73%) dropout; adjusted residual −0.3/0.3 | 77.27% | N/A | 22.73% |
| Ponce-Ramírez et al., 2024 (3rd intervention arm) [108] | N/A | Use app minimum 3x/week; progressive daily step target 7152→12520 steps over 10 weeks | 16/22 (72.73%) completed; 6/22 (27.27%) dropout; adjusted residual −0.9/0.9 | 72.73% | N/A | 27.27% |
| Ponce-Ramírez et al., 2024 (4th intervention arm) [108] | N/A | Use app minimum 3x/week; progressive daily step target 7152→12520 steps over 10 weeks | 16/22 (72.73%) completed; 6/22 (27.27%) dropout; adjusted residual −0.9/0.9 | 72.73% | N/A | 27.27% |
| Ramalho et al., 2025 [102] | 24 weekly Facebook videos/tasks + 24 weekly self-monitoring questionnaires + up to 6 monthly chat sessions (optional) | Engage weekly with Facebook group psychoeducational content and complete weekly self-monitoring questionnaire on the web app; request monthly chat sessions as needed (no explicit minimum interaction threshold stated, p. 6-7) | 85.51% (59/69) remained members of the APOLO-Teens Facebook group until the end of the 6-month intervention (attrition 14.49%); among the n=38 per-protocol completer subsample: 55.26% (n=21) viewed at least 12 of 24 weekly psychoeducational videos (M=12.50, SD=7.51); mean 7.68 (SD=6.23) of 24 weekly self-monitoring questionnaires completed; 23.7% (n=9) requested at least one of up to 6 monthly chat sessions (M=1.67, SD=0.71, range 1-3); usability questionnaire (n=35) rated the program highly-to-extremely useful/satisfying | 85.51% | 85.51% | 14.49% |
| Roche et al., 2024 [107] | 5 | Watch all 5 video modules within 1 week | 38/45 (84.4%) completed all 5 modules and follow-up knowledge/interest assessments; 7/45 (16%) completed baseline but did not complete all modules and were classified as dropout; up to 3 reminder emails sent to those with outstanding modules | 84.4% | N/A | 15.6% |
| Roche et al., 2024 (2nd intervention arm) [107] | 5 | Access all 5 online pamphlets/readings within 1 week | 33/39 (84.6%) completed all 5 modules and follow-up knowledge/interest assessments; 6/39 (15%) completed baseline but did not complete all modules and were classified as dropout; up to 3 reminder emails sent to those with outstanding modules | 84.6% | N/A | 15.4% |
| Seiterö et al., 2025 [109] | N/A | Encouraged to use according to individual needs and preferences; no fixed minimum stated | 58% (219/377) engaged with the intervention at least once; weekly screening completion 15% (57/377) in month 1 declining to 7% (26/377) in month 4; 12% (45/377) activated one or more SMS text-message programs | N/A | 58% | 35.28% |

^1^Proportion of participants who completed the entire intervention. ^2^Overall intervention adherence rate or mean adherence rate across all intervention components.

**Appendix S4. Synthesis Without Meta-analysis (SWiM) effect-direction table**

Direction is the predominant association with adherence across studies. Evidence type: S = statistical (inferential, p < .05), D = descriptive, Q = qualitative. PA = physical activity, Ob = obesity, Di = diet, To = tobacco, Al = alcohol, MC = multicomponent.

**Intervention-related factors**

|  | **Factor** | **Direction** | **Evidence** | **Domains** | **Consistency** |
| --- | --- | --- | --- | --- | --- |
|  | Personalisation / tailoring | Facilitator | S, D, Q | PA, Ob, Di, MC, To, Al | Consistent |
|  | User-friendly / intuitive design, usability | Facilitator | S, D, Q | All domains | Consistent |
|  | Reminders / prompts (well-timed; multiple prompts help under-engaged) | Facilitator | S, D | PA, Ob, Di, MC, To, Al | Consistent (timing/dose-dependent) |
|  | Goal-setting (flexible, adaptive) | Facilitator | D, Q | PA, Di, MC | Consistent (flexibility-dependent) |
|  | Gamification / competition / points | Facilitator | S, D, Q | PA, Ob, Di, MC, To, Al | Mostly consistent (competition can demotivate low performers; coercive use backfires) |
|  | Self-monitoring / tracking + feedback | Facilitator | S, D, Q | PA, Ob, Di, MC, Al | Mixed (downgraded: monitoring burden, null dose-response) |
|  | Human / coach / nurse / teacher / counselor support | Facilitator | S, D, Q | Ob, PA, To, Di, MC | Consistent (guided > unguided; optional support underused) |
|  | Parental / caregiver involvement | Facilitator | S, D, Q | Ob, MC, PA, Al | Consistent (caregiver competition only weakly motivating) |
|  | Content variety / quality (video, storylines, recipes) | Facilitator | S, D, Q | PA, Di, MC, To | Consistent |
|  | Technical difficulties (sync, login, notifications, platform blocks) | Barrier | D, Q | All domains | Highly consistent |
|  | Longer duration / engagement decline over time | Barrier | S, D, Q | PA, Di, MC, Al | Consistent |
|  | Information / education-heavy or over-long modules | Barrier (mixed) | D, Q | PA, Ob, Di, MC, Al | Mixed (overload direction now well-corroborated) |
|  | Poorly-timed / repetitive / excessive messages or surveys | Barrier | D, Q | PA, MC, Al, Di, To | Consistent |
|  | Social / peer support and connectedness | Facilitator (mixed) | S, D, Q | PA, MC, To, Di | Mostly consistent (in-person/relatable peers help; virtual-only weaker) |
|  | Extrinsic incentives (financial or academic credit) | Facilitator (mixed) | S, D | PA, MC, To, Di | Mostly consistent (short-term; gains fade after withdrawal; coercive use backfires) |
|  | Integration with familiar platform / clinical workflow / remote delivery | Facilitator | S, D | PA, To, MC | Consistent |

**User-related factors**

|  | **Factor** | **Direction** | **Evidence** | **Domains** | **Consistency** |
| --- | --- | --- | --- | --- | --- |
|  | Intrinsic motivation / perceived benefit / self-efficacy | Facilitator | S, D, Q | PA, Ob, MC, To, Al | Consistent |
|  | Higher education / health literacy / SES | Facilitator | S, D | Di, Al, MC | Consistent within domains |
|  | Lower baseline risk behaviour | Facilitator (mixed) | S, D | Al, To, MC, PA | Mixed (downgraded: higher-risk sometimes more engaged; engagement vs retention differ) |
|  | Female gender | Facilitator (domain-dependent) | S, D | Ob, Al, MC, To | Mixed across domains (contradictory even within To) |
|  | Younger age | Facilitator (domain-dependent) | S, D | Ob, Al, MC, PA, To | Mixed across domains (younger favours retention; older favours reuse) |
|  | Technical / access constraints (wifi, device, storage) | Barrier | D, Q | PA, MC, Di | Consistent |
|  | Time constraints / competing demands | Barrier | D, Q | PA, Ob, Di, MC, Al | Highly consistent |
|  | Lost motivation / lack of interest | Barrier | D, Q | PA, Ob, MC, To | Consistent |
|  | Depression / psychological distress | Barrier | S, D | Ob, Al, To | Consistent within domains (strengthened) |
|  | Minority / immigration background / marginalisation | Barrier (mixed) | S, D | MC, To, Al | Mixed across domains (barrier in To, facilitator in Al) |
|  | Illness burden / disease-related symptoms and clinical constraints | Barrier | D, Q | PA, MC | Consistent (in clinical populations) |
|  | Privacy / confidentiality and disclosure concerns | Barrier (mixed) | D, Q | To, MC, Di | Emerging (private/anonymous design can reassure) |
